# Phylogenomic analyses confirm a novel invasive North American *Corbicula* (Bivalvia: Cyrenidae) lineage

**DOI:** 10.7717/peerj.7484

**Published:** 2019-08-22

**Authors:** Amanda E. Haponski, Diarmaid Ó Foighil

**Affiliations:** Department of Ecology and Evolutionary Biology and Museum of Zoology, University of Michigan, Ann Arbor, MI, USA

**Keywords:** Clonal, Genomics, *Corbicula*, Form D, Lineage, ddRADseq, Invasive

## Abstract

The genus *Corbicula* consists of estuarine or freshwater clams native to temperate/tropical regions of Asia, Africa, and Australia that collectively encompass both sexual species and clonal (androgenetic) lineages. The latter have become globally invasive in freshwater systems and they represent some of the most successful aquatic invasive lineages. Previous studies have documented four invasive clonal lineages, Forms A, B, C, and Rlc, with varying known distributions. Form A (R in Europe) occurs globally, Form B is found solely in North America, mainly the western United States, Form C (S in Europe) occurs both in European watersheds and in South America, and Rlc is known from Europe. A putative fifth invasive morph, Form D, was recently described in the New World from the Illinois River (Great Lakes watershed), where it occurs in sympatry with Forms A and B. An initial study showed Form D to be conchologically distinct: possessing rust-colored rays and white nacre with purple teeth. However, its genetic distinctiveness using standard molecular markers (mitochondrial cytochrome *c* oxidase subunit I and nuclear ribosomal 28S RNA) was ambiguous. To resolve this issue, we performed a phylogenomic analysis using 1,699–30,027 nuclear genomic loci collected via the next generation double digested restriction-site associated DNA sequencing method. Our results confirmed Form D to be a distinct invasive New World lineage with a population genomic profile consistent with clonality. A majority (7/9) of the phylogenomic analyses recovered the four New World invasive *Corbicula* lineages (Forms A, B, C, and D) as members of a clonal clade, sister to the non-clonal Lake Biwa (Japan) endemic, *Corbicula sandai*. The age of the clonal clade was estimated at 1.49 million years (my; ± 0.401–2.955 my) whereas the estimated ages of the four invasive lineage crown clades ranged from 0.27 to 0.44 my. We recovered very little evidence of nuclear genomic admixture among the four invasive lineages in our study populations. In contrast, 2/6 *C. sandai* individuals displayed partial nuclear genomic Structure assignments with multiple invasive clonal lineages. These results provide new insights into the origin and maintenance of clonality in this complex system.

## Introduction

The clam genus *Corbicula* contains both sexual and asexual forms (see Lee et al., 2005 and references therein; Hedtke, Glaubrecht & Hillis, 2011; Pigneur et al., 2011, 2012, 2014), with the former restricted to fresh or brackish waters in their native range in the temperate/tropical regions of Asia, Africa, and Australia (Glaubrecht & Korniushin, 2003; Morton, 1986; Pigneur et al., 2014; Sousa, Antunes & Guilhermino, 2008). Asexual *Corbicula* lineages have been invading freshwater ecosystems across the globe for almost a century and are considered a major aquatic pest (summarized by Tiemann et al., 2017). Their first documented appearance outside of their native range occurred in western North America in the 1920s (summarized by Lee et al., 2005; Tiemann et al., 2017) and since then they have reportedly spread throughout North and much of South America (Beasley, Tagliaro & Figueiredo, 2003; Lee et al., 2005) and across Europe (Pigneur et al., 2011, 2014; Crespo et al., 2015; Peñarrubia et al., 2017), with Antarctica being the only continent with no known occurrences (Crespo et al., 2015; GBIF.org, 2019).

In the New World, studies have reported three invasive freshwater *Corbicula* lineages, two of which (Forms A and C) have also invaded European watersheds (Pigneur et al., 2014). Form A (also known as Form R in Europe; see Pigneur et al., 2014) is by far the most abundant New World invader occurring from Michigan to Patagonia (Lee et al., 2005). The New World distribution of Form C (Form S in Europe; see Pigneur et al., 2014) has been recorded only from southern South America (Ituarte, 1994; Lee et al., 2005). Form B is known solely from the New World occurring predominantly in the western United States but appears to be expanding its range eastward (summarized by Tiemann et al., 2017). A fourth invasive European lineage (Rlc) has yet to be reported in the New World (Pigneur et al., 2014).

**Figure 1:**
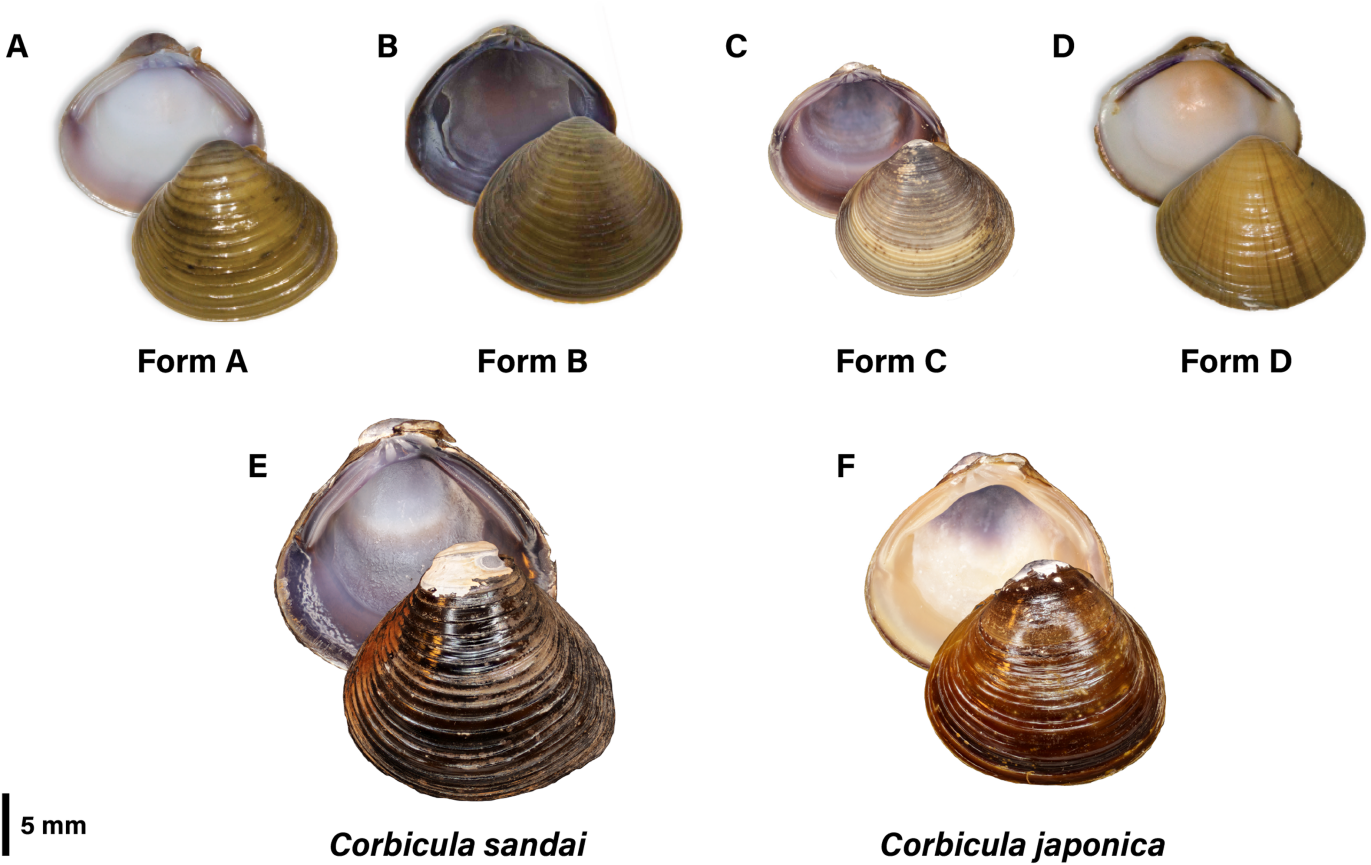
Photographic plate of the four invasive New World *Corbicula* Forms (A) A, (B) B, (C) C, and (D) D and the sexually reproducing (E) Lake Biwa endemic *C. sandai* and (F) estuarine *C. japonica* specimens genotyped in this study. Photos of Forms A, B, and D were taken by M. Jared Thomas and Danielle Ruffatto and Form C, *C. sandai*, and *C. japonica* by Taehwan Lee.

Forms A, B, and C are readily distinguishable by their shell phenotypes (see Fig. 1; see Lee et al., 2005; summarized by Tiemann et al., 2017). Shells of Form A are inflated and round to slightly pyramidal in shape with numerous concentric, evenly spaced and highly elevated ridges. The exterior is typically yellow or light to dark brown in color with the interior nacre highly polished and is characteristically white but may have suffusions of pink or light purple particularly around the teeth. Form B is round with ridges that are more closely spaced and less elevated than in Form A. Its exterior is typically dark olive to brown in color and its nacre and lateral teeth are both purple. A more detailed analysis of the differences between Forms A and B can be found in Britton & Morton (1986). The Form C morphotype has by far the finest external surface sculpture and the thinnest and least inflated shell of all three New World morphotypes. It is further distinguished from the co-occurring Form A morphotype by the latter’s prominent umbo, posterior rostrum, and lighter coloration (Ituarte, 1994).

Understanding the evolutionary relationships of these invasive *Corbicula* lineages has been complicated because of their unusual androgenetic reproductive mode. This is a rare form of clonal reproduction involving all male nuclear genome inheritance. In androgenetic *Corbicula* clams, an abortive first meiotic division leads to the ejection of the maternal nuclear genome and its replacement by the unreduced paternal pronucleus of the “fertilizing” sperm cell (Komaru et al., 1997; Konishi et al., 1998; Ishibashi et al., 2003; Lee et al., 2005; Hedtke et al., 2008; summarized by Pigneur et al., 2012, 2014). The only maternal genetic signature that persists in these asexually produced *Corbicula* clams is the egg mitochondrial (mt) genome. These clonal invasive Asian freshwater lineages are characterized by unreduced biflagellate sperm and come in diploid (Komaru et al., 1997; Komaru & Konishi, 1999; Park et al., 2000), triploid (Okamoto & Arimoto, 1986; Komaru et al., 1997; Komaru & Konishi, 1999; Park et al., 2000; Qiu, Shi & Komaru, 2001), and tetraploid (Qiu, Shi & Komaru, 2001) genomic iterations. In the New World, invasive populations of Forms A, B, and C also have been inferred to be androgenetic clones as they contain unreduced biflagellate sperm (Burch, Park & Chung, 1998; Lee et al., 2005; Hedtke et al., 2008; Pigneur et al., 2012, 2014). Moreover, triploidy (3*n* = 54) and a lack of complete meiotic metaphases has been documented for Forms A and B (Burch, Park & Chung, 1998; see Lee et al., 2005). Additionally, these invasive *Corbicula* lineages are hermaphroditic (see Komaru et al., 2012; McMahon, 1991; Ituarte, 1994) compared to their sexual counterparts that are dioecious. The presence of these characteristics means that a single asexual individual may potentially establish a new population, likely aiding their overall invasion success.

Previous studies have shown that the invasive New World clonal lineages are distinguishable using a variety of nuclear markers including allozyme loci (Hillis & Patton, 1982), ribosomal 28S and/or 18S DNA sequences (Lee et al., 2005; Hedtke et al., 2008), single copy genes (Hedtke, Glaubrecht & Hillis, 2011), and microsatellite loci (Pigneur et al., 2014). They are also distinct for most, but not all, populations in the widely used mt barcode marker cytochrome oxidase *c* subunit I (COI; Lee et al., 2005; Hedtke et al., 2008; Pigneur et al., 2014).

However, the phylogenetic relationships among the invasive clonal lineages worldwide are poorly supported in both nuclear and mt datasets (Lee et al., 2005; Hedtke et al., 2008; Hedtke, Glaubrecht & Hillis, 2011; Pigneur et al., 2012, 2014). There is evidence of hybridization between forms with heterozygous genotypes found in some specimens, e.g., heterozygous sequences were documented for nuclear 28S genotypes between clonal lineages B and C from Iguazu Falls in South America (Lee et al., 2005), Forms A and C in the Ebro River delta, Spain (Peñarrubia et al., 2017), and between Forms R and S from Northern Russia (Bespalaya et al., 2018) and Pigneur et al. (2014) found evidence of hybridization between Forms A and B in the Ohio River and between A and C in the River Seine using microsatellite loci. In addition, these asexual invasive *Corbicula* lineages appear capable, in at least some cases, of parasitizing the eggs of other co-occurring clones where the sperm of one clone “steals” the eggs, and thus the mitochondria, of another clone (Lee et al., 2005; Hedtke, Glaubrecht & Hillis, 2011; Pigneur et al., 2014; Tiemann et al., 2017). This apparent capacity for hybridization of nuclear markers (Lee et al., 2005; Pigneur et al., 2012, 2014; Peñarrubia et al., 2017; Bespalaya et al., 2018) and clonal capture of mt genomes complicates our ability to accurately interpret the evolutionary distinctiveness and origins of the invasive clonal lineages.

**Figure 2:**
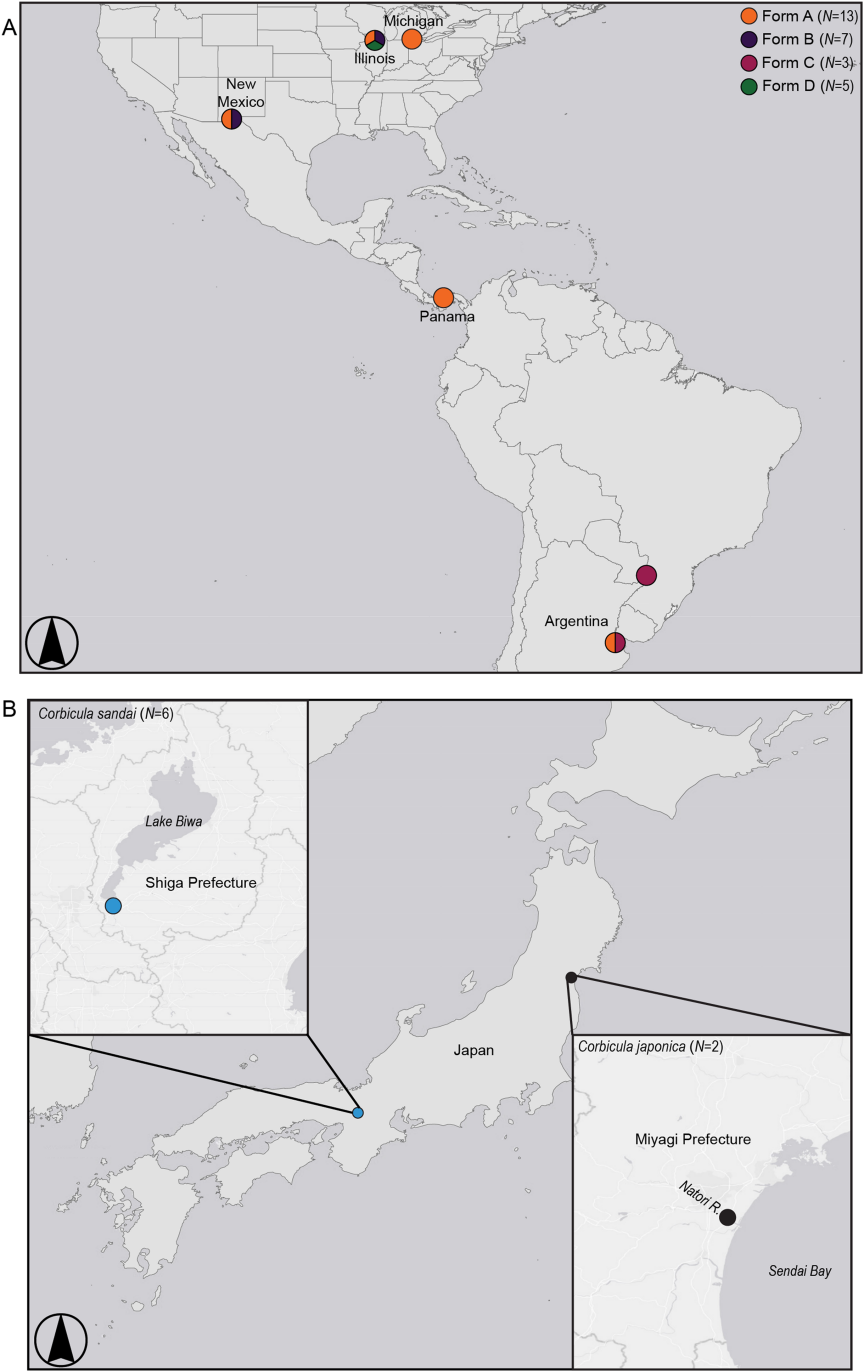
Map showing sampling locations for (A) invasive New World *Corbicula* Forms A, B, C, and D and (B) the sexually reproducing Lake Biwa endemic *C. sandai* and estuarine *C. japonica* specimens. See Table S1 for sampling location details and number of individuals per site. Map was created using ArcMap v10.6.1 (Esri, Redlands, CA, USA).

A recent article by Tiemann et al. (2017) identified a putative fifth invasive lineage (fourth to the New World), “Form D” (see Fig. 1) from a single site in the Illinois River sympatric with Forms A and B (Fig. 2A; see Tiemann et al., 2017). It has a highly distinctive shell phenotype, with shells being pyramidal in shape with weakly elevated ridges, a yellowish-brown exterior with fine rust-colored rays radiating out from the umbo. The interior of the shell has a creamy white nacre with purple lateral teeth (see Fig. 1). The Form D specimens also differed from co-occurring Forms A and B in their nuclear 28S ribosomal DNA genotype (differing by three to seven mutational steps) but shared a mt COI haplotype with sympatric Form A clams. Tiemann et al. (2017) proposed that this cyto/nuclear disjunction may have resulted from *in situ* androgenetic capture of Form A eggs by Form D sperm, an apparently common phenomenon among invasive co-occurring *Corbicula* lineages (see Lee et al., 2005; Hedtke et al., 2008; Pigneur et al., 2014).

Although the Form D 28S genotype is distinct from all invasive *Corbicula* lineages characterized using direct sequencing (Tiemann et al., 2017), it matched a minority cloned genotype (i.e., individual DNA sequences inserted into bacterial plasmids) obtained from two Form B individuals (1/6 and 1/8 cloned genotypes, respectively) sampled in Texas (Hedtke et al., 2008) and one of three cloned genotypes obtained from a Form B-like individual in Japan (Komaru et al., 2012; Komaru, Yamada & Houki, 2013). The cloning of amplified gene fragments by these studies allowed characterization of both rare and predominant genotypes, but this approach can be prone to sequencing errors (reviewed by McInerney, Adams & Hadi, 2014). Although nuclear genomes contain large copy numbers of ribosomal genes, these copies typically show very modest levels of intraspecific variation presumably due to concerted evolution and exceptions stem either from gene duplication events or reticulation processes (Hillis & Dixon, 1991). It is possible that the minority presence of this 28S genotype in Form B (Texas) and Japanese (Form B-like) populations stems from reticulation processes, but in the Illinois River Form D samples this genotype lacked heterozygous or paralogous nucleotide profiles in the direct sequences and showed no evidence for a reticulate origin.

Thus, Tiemann et al. (2017) concluded that Form D was likely a new clonal invasive lineage based on its novel shell phenotype and its distinctive nuclear 28S sequences to sympatric Forms A and B, but that this needed to be confirmed with a more comprehensive molecular dataset. To address this question, we performed the first phylogenomic study of the genus *Corbicula*, sequencing 1,699–30,027 nuclear genomic loci with the double digest restriction-site associated sequencing (ddRADseq) method of Peterson et al. (2012).

## Materials and Methods

### Sampling

We sampled five individuals each of the three Illinois River Forms A, B, and putative D (Table S1; Fig. 2). To place these 15 individuals into a broader phylogenomic framework, we included congeners from the invasive range in North and South America representing all four reported New World morphotypes (Forms A, B, C, and D; Table S1; Fig. 2), as well as two sexual Asian *Corbicula* species; the freshwater Lake Biwa endemic *C. sandai* (*N* = 6) and the estuarine *C. japonica* (*N* = 2; Table S1; Fig. 2). Individuals of the four New World morphotypes and CSA1 for *C. sandai* were previously analyzed for mt COI and 28S in Lee et al. (2005) and Tiemann et al. (2017).

### ddRADseq data collection and bioinformatics

The DNA of the 36 *Corbicula* specimens genotyped in this study was extracted and their quantities assessed following the protocols of Haponski, Lee & Ó Foighil (2019). We targeted ~200 ng of DNA for library preparation, any individuals with DNA quantities less than this were re-extracted. ddRADseq libraries then were prepared and followed the protocols of Peterson et al. (2012) and Haponski, Lee & Ó Foighil (2019). Prepared ddRADseq libraries were submitted to the University of Michigan’s DNA sequencing core (https://brcf.medicine.umich.edu/cores/dna-sequencing/) and run in two different lanes using 125 bp paired-end sequencing on Illumina NextSeq using the mid-range 300 cycle and a HiSeq 2500.

Sequence quality first was assessed using Fastqc v.0.11.5 (Andrews, 2018) and showed the presence of Illumina adapters in the HiSeq 2500 sequencing lane and Phred quality scores across both lanes ranging from 14 to 38. Raw sequences then were deposited on the Flux high computing cluster at the University of Michigan’s Center for Advanced Computing for further processing and analyses. The alignment-clustering algorithm in ipyrad v.0.7.17 (Eaton, 2014; Eaton & Overcast, 2019) was used to process and identify homologous ddRADseq loci using settings from Haponski, Lee & Ó Foighil (2019). Processed reads were clustered and aligned across individuals, filtered for paralogs, and finally concatenated into consensus loci at 85%, 90%, and 95% similarity *de novo* in ipyrad. We also varied the minimum number of individuals required for a consensus homologous locus to be retained in the final dataset with a final filtering step that removed any loci not recovered across (1) 75% (*N* = 27), (2) 50% (*N* = 18), or (3) 25% (*N* = 9) of individuals. Output files for these final nine concatenated datasets were exported for further downstream analysis and file conversion where needed.

### Determining the number of clonal *Corbicula* multi-locus genotypes

The potential presence of clonal *Corbicula* clams can be problematic for traditional phylogenomic and population genomic analyses since clonal populations typically violate many assumptions of the methods (see Grünwald et al., 2017) and identical sequences should be pruned prior to constructing phylogenetic trees (see Harrison & Langdale, 2006). Prior to performing our analyses on the 28 purported invasive clonal *Corbicula* individuals (Forms A, B, C, and D), we performed a clone correction so that only a single individual represented each of our unique multi-locus genotypes (MLG). This ensured that our corrected clonal lineages approximated the behavior of sexual populations for our analyses (see Grünwald et al., 2017). Clone correction was conducted using the package poppr v2.8.1 (Kamvar, Tabima & Grünwald, 2014; Kamvar, Brooks & Grünwald, 2015; Kamvar et al., 2018) in R v3.3.3 (R Development Core Team, 2017) following the poppr tutorial and using default settings (Kamvar et al., 2018). Poppr determined that each of our 28 *Corbicula* clonal individuals was a unique MLG. Thus, we include all 28 individuals representing Forms A (*N* = 13), B (*N* = 7), C (*N* = 3), and D (*N* = 5) in all phylogenomic and population genomic analyses.

### Phylogenomic analyses of *Corbicula* clams

To determine phylogenomic relationships among the 36 *Corbicula* clams, we used methods detailed in Haponski, Lee & Ó Foighil (2019). We analyzed the nine concatenated ddRADseq alignment files of homologous loci using maximum likelihood (ML) in RAxML v8.2.8 (Stamatakis, 2014). We specified the two *C. japonica* samples as the outgroup. Analyses utilized the general time reversible model (GTR; Lanave et al., 1984) and included invariable sites and a gamma distribution. Support for nodes was determined from 100 fast parametric bootstrap replications. The nine resulting trees showed congruent phylogenomic relationships and similar support values among major *Corbicula* clades (see Fig. S1). Since these relationships were largely consistent across the nine datasets (see Results), we then selected the 90% similarity threshold with 75% of individuals included (90–75 hereafter) for all remaining analyses as it had an intermediate number of loci (2,245), intermediate similarity threshold (90%), and had at least 27/36 individuals (75%) present in every locus.

In addition to the RAxML analyses, we conducted a Bayesian analysis on the concatenated 90–75 alignment in the parallel version of MrBayes v3.2.6 (Ronquist et al., 2012). We again specified the two *C. japonica* individuals as the outgroup. Bayesian analyses followed Haponski, Lee & Ó Foighil (2019) and included the GTR model with invariable sites and a gamma distribution and used a Metropolis-coupled Markov chain Monte Carlo (MC^3^) approach and ran for 4,000,000 generations, with sampling every 100. Two analyses were performed each with four separate chains run simultaneously. Stationarity and burn-in period for the MC^3^ was determined by plotting log likelihood values for each generation. The first 25% of the generations, trees, and parameter values sampled were discarded as burn-in. The runs were considered to have reached convergence when the average split standard deviation was <0.01, the potential scale reduction factor was between 1.00 and 1.02, and log likelihood plots appeared as white noise (Ronquist, Huelsenbeck & Teslenko, 2011). A 50% majority rule consensus tree was based on the remaining generations, whose branch support was determined from the posterior probability distribution (Holder & Lewis, 2003) in MrBayes.

**Figure 3:**
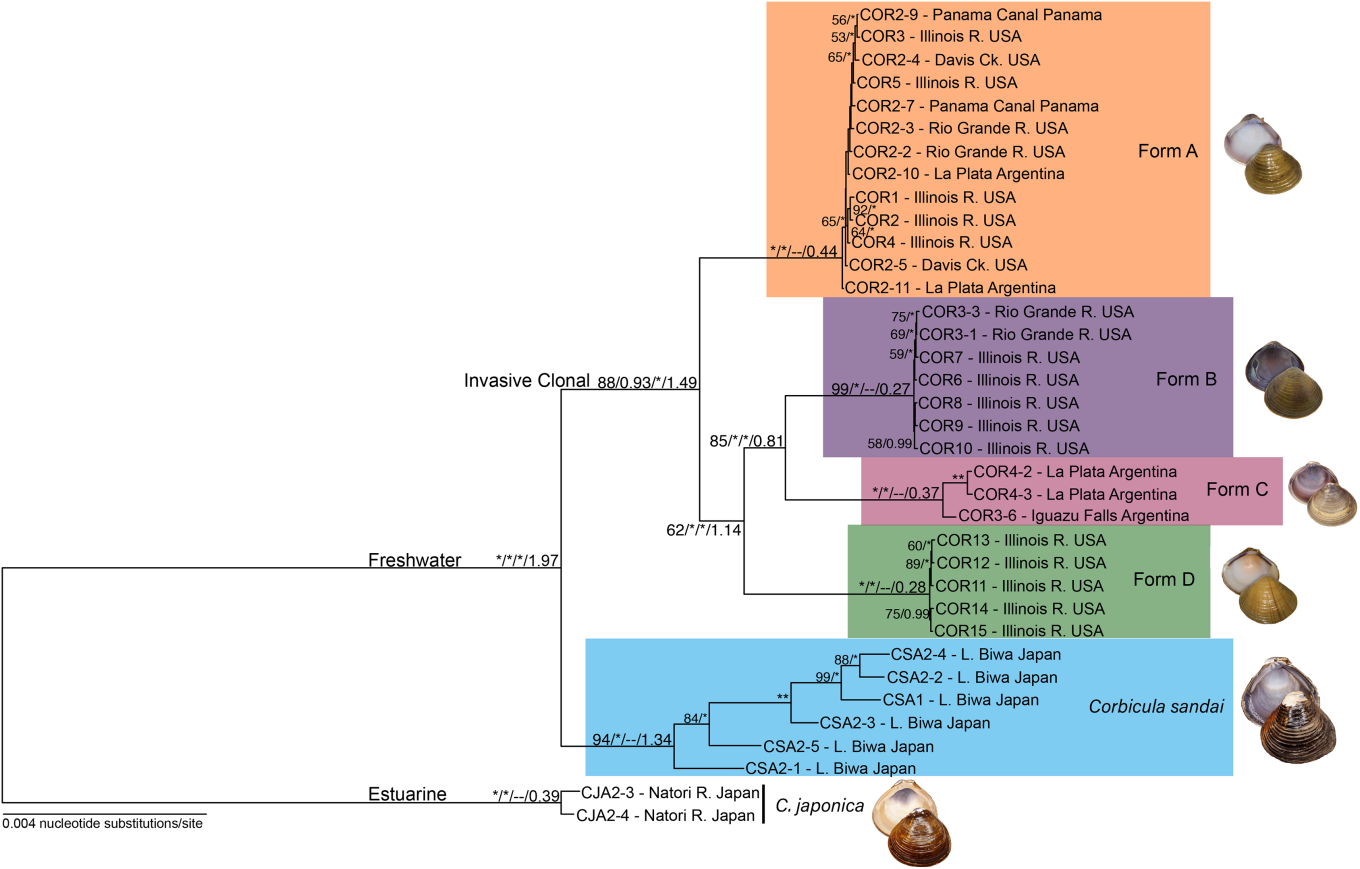
Maximum likelihood phylogenomic tree depicting relationships among the four invasive New World *Corbicula* forms and the sexually reproducing *C. sandai* for the 2,245 locus 90% similarity threshold clustering across 75% of individuals. Tree was rooted with two individuals of *C. japonica*. Values on tree nodes indicate maximum likelihood bootstrap/posterior probability supports/speciation event supports from mPTP/BEAST age estimates. * Denotes bootstrap and posterior probability supports of 100% and 1.00, respectively. Photos of Forms A, B, and D were taken by M. Jared Thomas and Danielle Ruffatto and Form C, *C. sandai*, and *C. japonica* by Taehwan Lee.

We assessed the phylogenomic distinctiveness of each of the *Corbicula* clades recovered in the RAxML and Bayesian analyses using the multi-rate Poisson tree processes (mPTP) model (Kapli et al., 2017). In comparison to other methods testing species limits mPTP requires a gene tree estimation as input but with branch lengths proportional to the amount of genetic change and tends to provide better estimates when interspecific distances are small (Zhang et al., 2013; Kapli et al., 2017). Although this method was designed for single-locus data, it is often applied to concatenated multi-locus data (reviewed by Luo et al., 2018). The analysis was performed using the concatenated 90–75 2,245 loci ML tree from RAxML (see Fig. 3) in mPTP v0.2.4 (Kapli et al., 2017). First, we calculated the minimum branch lengths (0.0000431598) and then performed MCMC sampling to assess the confidence in the ML tree. mPTP was run using the *-multi* option to incorporate differences in rates of coalescence among clades. We ran the MCMC analysis using the three different starting delimitations available, null, random, and ML, and each analysis comprised 10 independent runs of 100,000,000 generations, sampling every 1,000 with the first 2,000,000 generations discarded as burn-in. Convergence of the null, random, and ML MCMC analyses then was assessed via the plot of generation vs. log-likelihood. All three starting delimitations arrived at the same topologic solution with supports of 0.98 ± 0.00002 for each.

### Divergence time estimation of *Corbicula* lineages

Comparative divergence time estimates among supported *Corbicula* lineages from the ML, Bayesian, and mPTP analyses were evaluated using BEAST v.2.5.1 (Bouckaert et al., 2014). We used the python code provided by Bongaerts (2018; vcf_single_snp.py) to first convert our variant call format (VCF) file from ipyrad containing all SNPs for the 90–75 dataset to randomly select a single SNP per locus. This dataset totaled 2,175 SNPs due to the presence of ~70 monomorphic loci (2,245 loci in the concatenated dataset). We then used PGDSpider v2.1.1.0 (Lischer & Excoffier, 2012) to convert the 2,175 single SNP VCF file to a nexus file. The BEAST XML file was generated using BEAUti incorporating the GTR nucleotide substitution model, a gamma distribution, and invariant sites, and using a relaxed molecular clock that assumed a lognormal distribution with the Yule speciation process (Gernhard, 2008) as a tree prior. Two separate BEAST runs were conducted, each with a chain length of 50,000,000 generations, and parameters sampled every 100 generations. We calibrated the phylogeny using the estimated age of Lake Biwa (~4 million years ago; mya; Yokoyama, 1984; Kawabe, 1989, 1994; Tabata et al., 2016) for the endemic *C. sandai*. This node was set using a log-normal distribution and a mean of 0.6 and a standard deviation of 0.35, which gave the age distribution an upper bound of ~4–5 mya. Analyses were viewed in Tracer v1.71 (Rambaut et al., 2018) and were considered to have reached stationarity when the effective sample size values were over 200 and traces appeared as white noise.

### Population genomic analyses of *Corbicula* lineages

For the population genomic analyses, we used the 2,175 single SNP dataset created for the BEAST analyses and converted it to the Genepop format in PGDSpider. Each of the four invasive *Corbicula* lineages and the endemic sexually reproducing *C. sandai* were tested for conformance to Hardy–Weinberg equilibrium (HWE) expectations in the Genepop package (Rousset, 2008) in R prior to analyses. Each of the HWE tests was run for an MCMC chain of 10,000, 1,000 batches, and 10,000 iterations in the Genepop package. Significance levels of all tests were adjusted with the sequential Bonferroni correction (Rice, 1989).

We also calculated observed (*H*_O_) and expected (*H*_E_) heterozygosities and the inbreeding coefficient (*F*_IS_) in Genepop to evaluate whether the invasive *Corbicula* lineages exhibited the typical genetic characteristics; i.e., low genetic diversity, excess of heterozygotes (see Stoeckel et al., 2006) observed for clonal populations. We calculated these values for a reduced dataset that removed any loci with missing data (*N* = 113/2,175 loci retained) and excluded the three Form C individuals due to sampling only two sites, with the individual (COR3–6) from one site having far fewer loci. We compared the diversity values of Form A, B, and D individuals to those for the sexually reproducing *C. sandai*. Significance of the values was compared using analysis of variance (ANOVA) followed by a Tukey’s test in *R*.

Using the two clustering approaches outlined in Haponski, Lee & Ó Foighil (2019), we examined the invasive *Corbicula* lineages and *C. sandai* for further population sub-division with the Bayesian based Structure v2.3.4 (Hubisz et al., 2009; Pritchard, Stephens & Donnelly, 2000) and discriminant analysis of principal components (DAPC; Jombart, Devillard & Balloux, 2010) analyses. As input for the former method we converted the 90–75 single SNP dataset to the Structure format using PGDSpider and removed any loci consisting entirely of missing data or non-polymorphic SNPs. The final dataset contained 2,175 SNPs.

A total of six Structure analyses were performed: the full dataset of 34 samples, for each of the invasive clusters (*N* = 4 analyses) and for *C. sandai*. For the full dataset of 34 samples, we varied the *K*-values from 1 to 8 (the number of well-supported clades in the phylogenomic trees (5) plus three) since we had sampled numerous locations across the New World (Table S1). Subsequent Structure analyses for each of the invasive lineages (Forms A, B, C, and D) then were run with the number of *K*-values ranging from *K* = 1 to the number of sampling locations plus one for each lineage. In the case of the five Form D individuals from the Illinois River we ran the analysis up to *K* = 3 to ensure all population genetic variation was recovered. Parameters for each of the Structure analyses followed Haponski, Lee & Ó Foighil (2019) performing 10 independent runs for each *K* with a burn-in length of 150,000 replicates followed by 500,000 generations. Stationarity and the optimal *K* were assessed using the Δ*K* method of Evanno, Regnaut & Goudet (2005) in the web-based StructureHarvester (Earl & Vonholdt, 2012) and posterior probabilities (Pritchard, Stephens & Donnelly, 2000) in Clumpak v1.1 (Kopelman et al., 2015). Results from Structure runs then were visualized using Distruct v1.1 (Rosenberg, 2004) in Clumpak.

We implemented the DAPC analyses (Jombart, Devillard & Balloux, 2010) via the adegenet package (Jombart, 2008) in R. DAPC analyses also followed an iterative approach with the initial run consisting of all 34 *Corbicula* specimens, and then five subsequent runs for the four invasive clusters (Forms A, B, C, and D) and for *C. sandai*. The Genepop formatted file used for HWE and genetic diversity calculations was used as input into DAPC. We first used the *K*-means clustering of principal components (PC) to identify groups of individuals (see Jombart, Devillard & Balloux, 2010) and determined the optimal number of PCs to maintain using the *optim.a.score* command. This showed one PC for the 34 individual dataset, however we maintained two (Fig. S2). The Bayesian information criterion was uninformative for determining the number of clusters as it did not show the characteristic profile (see Jombart & Collins, 2015; Fig. S2). The *K-*means clustering for each of the invasive lineages (A, B, C, and D) and *C. sandai* also did not recover any additional variation within these clusters. Relationships among the clusters for the 34 individual analysis were determined by plotting the first two PCs of the DAPC.

We also tested for admixture among the four invasive *Corbicula* lineages and the *C. sandai* samples using Structure assignments and threepop (*f*_3_) tests (Reich et al., 2009) as in Haponski, Lee & Ó Foighil (2019). Briefly, the threepop test is formulated as *f*_3_(1;2,3) and compares whether population 1 has inherited a history of admixture using populations 2 and 3 as reference points (see Reich et al., 2009). A significantly negative value implies that population 1 is admixed (see Reich et al., 2009; Pickrell & Pritchard, 2012). We grouped the *Corbicula* specimens into five groups; each of the invasive Forms (A, B, C, or D) and *C. sandai* based on results from the phylogenomic trees, Structure, and DAPC analyses. We calculated the *f*_3_ test-statistic values in the program TreeMix v1.13 (Pickrell & Pritchard, 2012). Input files for TreeMix were created using the python code vcf2treemix.py (Silva, 2018) and all possible *f*_3_ comparisons were run using blocks of 100 SNPs. Significance of *Z*-score values then was assessed in *R*.

### Data archiving

The raw data for each of the 36 *Corbicula* individuals from the Illumina HiSeq were deposited in NCBI’s Sequence Read Archive (Accession # PRJNA526860). Parameter files used to generate the 85%, 90%, and 95% threshold datasets for 75%, 50%, and 25% of taxa from ipyrad were deposited in the Dryad Digital Repository (DOI 10.5061/dryad.4395r55) along with all data matrices used to construct the ML and Bayesian trees and for the BEAST, Structure, and DAPC analyses. We also deposited the .fasta alignment and newick tree file used for the mPTP analyses and the single SNP .vcf file used to generate the matrices for BEAST, Structure, and DAPC analyses. All relevant data also are available from the authors.

## Results

### Summary of ddRADseq data

Illumina sequencing returned raw read numbers ranging from 125,580 to 5,883,062 per specimen across the 36 corbiculid samples, with 12 individuals having fewer than 1,000,000 reads (Table S2). We recovered 1,699–30,027 inferred homologous nuclear genomic loci across the three similarity thresholds (85%, 90%, 95%) and three minimum taxon coverages (25%, 50%, 75%; totaling nine ddRADseq datasets). Across the 36 individuals, the invasive *Corbicula* morphotypes had higher numbers of inferred homologous loci (372–24,443) compared to the sexually reproducing species; the freshwater *C. sandai* (289–8,295) and estuarine *C. japonica* (26–229). In general, the numbers of inferred homologous loci for the four invasive *Corbicula* forms were similar across samples, the different similarity thresholds (85%, 90%, and 95%) and taxon coverages (75%, 50%, and 25%; Table S3) with the exception of one Form C individual (COR3–6) that had by far the lowest number of identified loci (372–1,297) compared to the other invasive clams (1,116–24,443). Further details on sequencing depth of coverage and locus trends across the datasets are included in Appendix 1 and summarized in Tables S2 and S3.

### Phylogenomic distinctiveness of Illinois River Form D

Our phylogenomic trees and mPTP analyses consistently showed five distinct freshwater *Corbicula* lineages; four New World invasive Forms (A, B, C, and D) and the sexually reproducing Lake Biwa endemic *C. sandai*. Notably, Form D sampled from the Illinois River is a distinct *Corbicula* lineage with high support (~98–100% and 1.00 posterior probabilities; Fig. 3; Figs. S1 and S3). Not only were Form D individuals genomically distinctive from sympatric Forms A and B sampled from the Illinois River, but they were distinct from all other *Corbicula* clams included in our analyses. Eight of the nine phylogenomic analyses consistently and robustly recovered a clade containing the four invasive lineages each corresponding to one of the four described morphotypes (A, B, C, and D) with high support (Fig. 3; Figs. S1 and S3). The one exception was the 85% similarity threshold analysis that included 75% of individuals (Fig. S1C). In this topology, the Form A clade collapsed onto the freshwater *Corbicula* stem node and, although the other freshwater lineages were recovered as monophyletic crown clades, there was very little support for internal nodes (Fig. S1C). The mPTP analyses also supported the identification of four phylogenomically distinct invasive lineages, Forms A, B, C, and D (Fig. 3; Fig. S3). Additionally, individuals of the sexually reproducing *C. sandai* endemic to Lake Biwa were consistently recovered as a distinct *Corbicula* lineage in all phylogenomic trees and mPTP analyses (Fig. 3; Figs. S1 and S3).

In all analyses *C. sandai* and the four invasive lineages formed a highly-supported (100% and 1.00 posterior probability) freshwater clade (Fig. 3; Figs. S1 and S3). In the 90–75 dataset depicted in Fig. 3, Form A, B, C, and D lineages clustered together in a well-supported invasive freshwater clade (88% and 0.93 posterior probability, 100% mPTP support; Fig. 3; Figs. S1 and S3). However, the relationships among the four invasive lineages were not well resolved with different datasets showing different topologies (Fig. 3; Fig. S1) and the most common topology (6/9 datasets) being (((B, C), D), A) (Fig. 3; Fig. S1). *C. sandai*, the Lake Biwa endemic, was robustly sister to this invasive clade in 6/9 phylogenomic analyses (Fig. 3; Fig. S1), the exceptions being the 85% similarity threshold with 75% of individuals (Fig. S1C), 85% including 50% (Fig. S1D), and 95% with 25% (Fig. S1I) where it placed among the invasive lineages. To determine if this variability was driven by insufficient loci, we removed individuals with the lowest number of recovered loci (invasive Form C individual COR3–6 and four *C. sandai* clams: CSA2–2, CSA2–3, CSA2–4, and CSA2–5) and redid the phylogenomic analyses for the three exceptions. *C. sandai* was recovered as the sister to the invasive freshwater clade for the 85–50 dataset (Fig. S1E). For the 85–75 (Fig. S1C) and 95–25 (Fig. S1I) datasets, the topology remained unchanged.

### Divergence time estimations of invasive *Corbicula* forms and *C. sandai*

According to the Lake Biwa calibrated BEAST divergence time estimates, the last common ancestor (LCA) of the freshwater lineages (Forms A, B, C, and D and *C. sandai*) was dated to ~1.974 mya and the LCA of the four invasive lineages (Forms A, B, C, and D) to ~1.49 mya (Fig. 3; Table 1). Among the crown clades, *C. sandai* was the oldest (~1.341 mya) compared to the much younger dates for the invasive lineages (~0.439–0.272 mya; Fig. 3; Table 1).

**Table 1:** Divergence time estimations for the four invasive New World *Corbicula* lineages and the sexually reproducing *C. sandai* for each of the nodes of the 90–75 RAxML tree (see Fig. 3).

Tree node	Age (my)	95% HPD
Form A	0.439	0.059–1.013
Form B	0.272	0.028–0.664
Form C	0.370	0.039–0.826
Form D	0.280	0.021–0.697
*C. sandai*	1.341	0.543–2.306
*C. japonica*	0.385	0.006–1.039
Forms B and C	0.807	0.191–1.627
Forms B, C, and D	1.139	0.291–2.268
Clonal (Forms A, B, C, and D)	1.492	0.401–2.955
Freshwater (*C. sandai*, Forms A, B, C, and D)	1.974	0.640–3.782

**Note:**

Values include the estimated age of the node in millions of years (my) and the 95% highest posterior density (HPD) interval.

### Population genomic Structure

**Figure 4:**
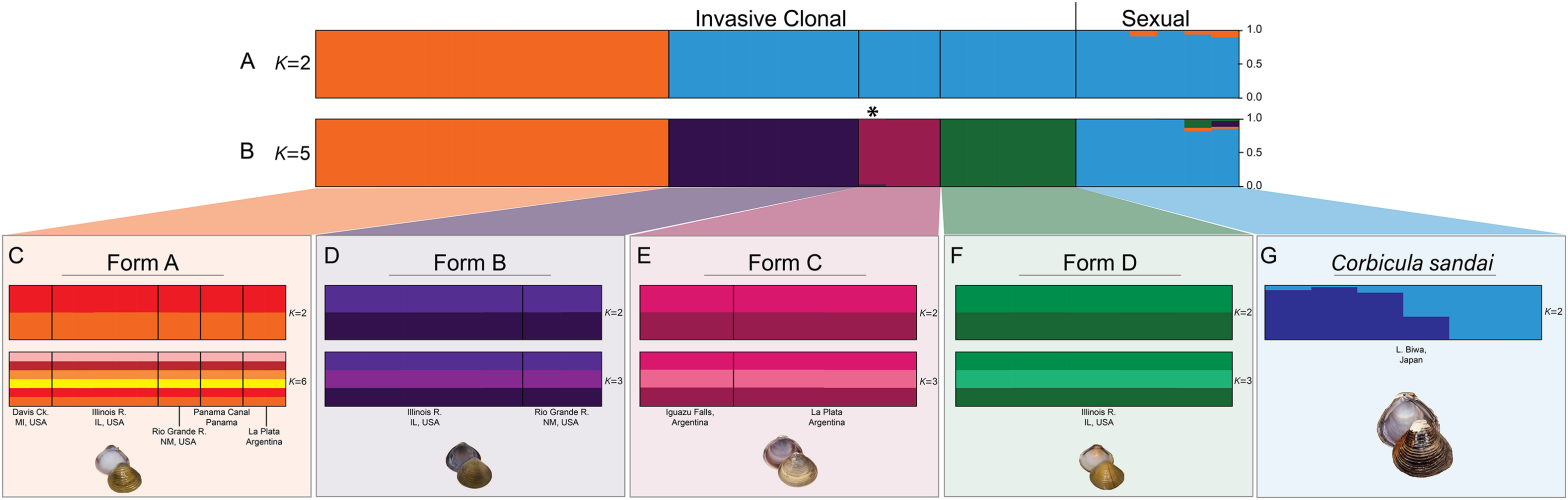
Structure bar graphs showing the most likely assignment of the 34 *Corbicula* individuals for (A) *K* = 2 and (B) *K* = 5, (C) Form A, (D) Form B, (E) Form C, (F) Form D, and (G) *C. sandai* based on ∆*K* (see Fig. S4). Structure analyses used a single SNP per locus (totaling 2,175 SNPs) for each individual and each vertical bar represents an individual clam. Labels on Structure graphs indicate the sampling locations for each individual (see Table S1). In (B) * indicates Form C individual COR3–6 from Iguazu Falls. Photos of Forms A, B, and D were taken by M. Jared Thomas and Danielle Ruffatto and Form C, *C. sandai*, and *C. japonica* by Taehwan Lee.

**Figure 5:**
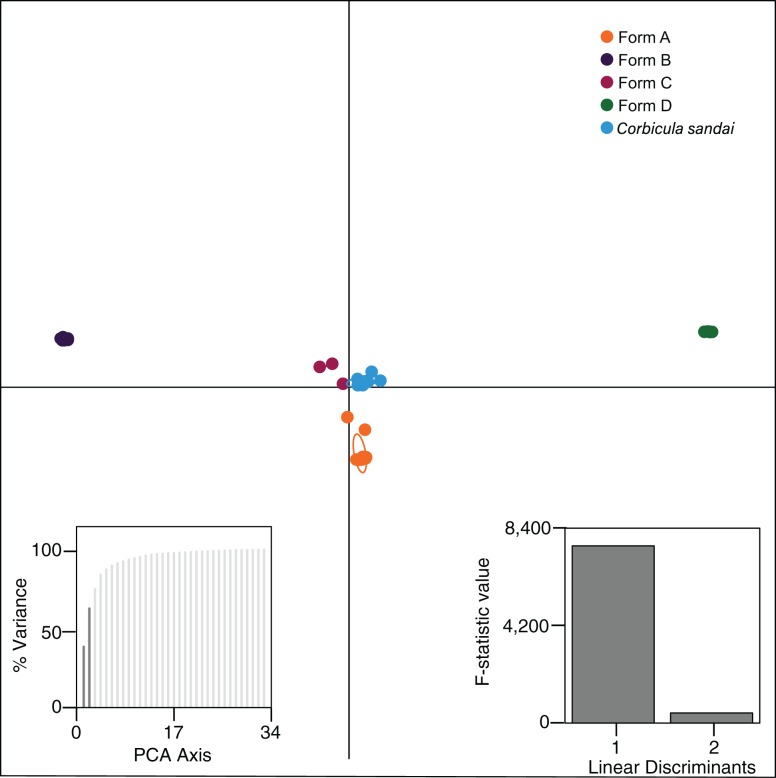
Results from the discriminant analysis of principal components (DAPC; Jombart, Devillard & Balloux, 2010) for the four invasive *Corbicula* forms and the sexually reproducing *C. sandai*. Individuals are represented as dots with 95% confidence intervals surrounding them. Clusters are color coded to match those recovered by the Structure analyses (Fig. 4).

Our Structure analyses indicated that the genomic variation was hierarchically partitioned as indicated by the Δ*K* and posterior probabilities (Fig. S4). The first-level identified just two population clusters (*K* = 2; Fig. 4A) with Form A individuals identified as one population and all remaining *Corbicula* samples, including Forms B, C, D, and *C. sandai* as another. The next level recognized five distinct population clusters distinguishing each of the four invasive *Corbicula* Forms (A, B, C, and D) and the sexually reproducing *C. sandai* as separate groups (Fig. 4B), similar to the phylogenomic analyses. Likewise, the DAPC analysis supported the recognition of five distinct clusters corresponding to the four invasive Forms and the sexually reproducing *C. sandai*. The DAPC analysis also showed a closer clustering of Forms A, C, and *C. sandai* (Fig. 5).

Notably, both analyses supported the genomic distinctiveness of Form D specimens from all other *Corbicula* clams sampled with 100% self-assignment to their genetic cluster in Structure analyses (Fig. 4) and a tightly formed cluster in the DAPC analysis (Fig. 5). Likewise, individuals identified as invasive Forms A, B, and C had 100% self-assignment in the Structure (Fig. 4B; Table S4) and DAPC (Fig. 5) analyses, with the exception of one individual. Form C individual COR3–6 from Iguazu Falls Argentina showed some potential admixture with Form B (~2% assignment; purple) in the Structure analysis (Fig. 4B; Table S4) and in the DAPC analysis it assigned to the *C. sandai* cluster (Fig. 5). Two of the six *C. sandai* individuals also showed evidence of admixture with the invasive forms exhibiting ~83% self-assignment in the Structure analyses. Individual CSA2–5 had ~11% assignment to Form D and ~5% to Form A and CSA2–1 had ~9% to Form B and ~3% each to Forms A and C (Fig. 4B; Table S4). Overall, the four invasive *Corbicula* forms and sexually reproducing *C. sandai* lineages showed very limited evidence of admixture based on the Structure and DAPC results. Similarly, the *f*_3_ admixture tests recovered no evidence of admixture among the five genomic *Corbicula* lineages.

We also analyzed the population structure within each of the five *Corbicula* lineages recovered in the phylogenomic, Structure, and DAPC analyses. The four invasive *Corbicula* forms showed no additional population separation (see Figs. 4C–4F; Fig. S5). The Δ*K* and posterior probabilities from Structure supported a single population (*K* = 1) for each of the invasive lineages as evidenced by their bar graphs. Regardless of the *K* specified, individuals equally assigned to each color indicating no structuring. Notably, Form A whose samples spanned the New World from North (Davis Ck., MI, USA) to South (La Plata, Argentina) America showed only a single population across this broad geographic range (Fig. 4C; Fig. S5). In contrast, *C. sandai* samples exhibited some genomic variation with Structure supporting two populations within the Lake Biwa specimens examined (Fig. 4G).

### Genomic diversity of invasive *Corbicula* forms and *C. sandai*

**Figure 6:**
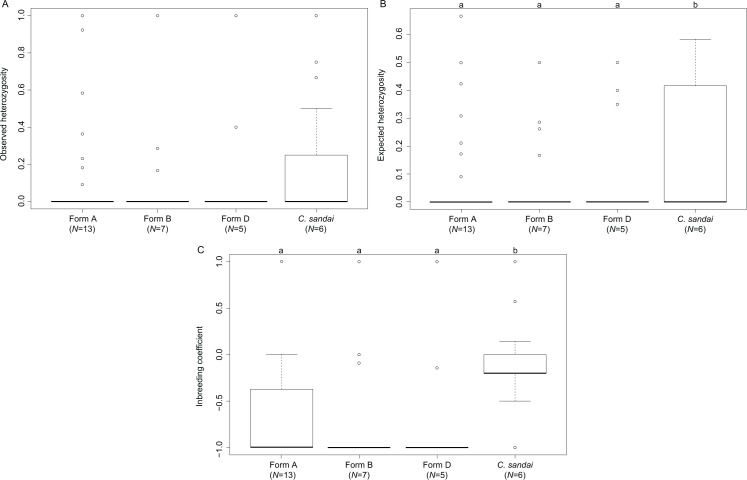
Boxplots showing diversity values for each of the invasive New World *Corbicula* forms and *C. sandai*. (A) Observed and (B) expected heterozygosity and (C) the inbreeding coefficient (*F*_IS_). Lowercase letters above the boxes indicate results from the Tukey’s test.

The invasive *Corbicula* Forms A, B, and D exhibited significant deviations from HWE (A, *p* < 0.0001; B, *p* < 0.0001; D, *p* < 0.0001) whereas the sexually reproducing *C. sandai* conformed to HWE expectations (*p* = 1.000). Genomic diversity calculations showed low observed heterozygosity levels among invasive Forms A, B, and D and sexually reproducing *C. sandai* individuals (ANOVA *F* = 0.280, *p* = 0.839; Table S1; Fig. 6). Invasive Forms A, B, and D also had low expected heterozygosity values whereas *C. sandai* had significantly higher levels (ANOVA *F* = 4.787, *p* = 0.003). The inbreeding coefficient (*F*_IS_) indicated significant excess of heterozygotes for all of the invasive forms with values close to −1 that significantly differed (ANOVA *F* = 14.09, *p* < 0.001) from *C. sandai* clams that had the only positive value (0.185 ± 0.077) in the dataset (Table S1; Fig. 6).

## Discussion

Our genomic results confirmed that the recently discovered Illinois River Form D population (Tiemann et al., 2017) represents a novel invasive New World *Corbicula* lineage. It formed a highly supported monophyletic clade in all analyses, distinguishing it from not only the sympatric Illinois River Form A and Form B samples but also from all other genotyped invasive New World congeners and from two non-invasive Asian congeners. These findings increase the number of known invasive New World *Corbicula* lineages to four: the previously described Forms A, B, and C (Lee et al., 2005) and the newly recognized Form D (Tiemann et al., 2017). This corroborates comprehensively the cyto/nuclear disjunction (mt COI and nuclear 28S) reported by Tiemann et al. (2017) and further supports the hypothesis of androgenetic egg capture by Form D of Form A mitochondria in the Illinois River population. There is still much we do not know about the invasive Form D lineage. Documenting the distribution of Form D is a pressing concern as the Illinois Natural History survey has already found Form D individuals at additional sites along the Illinois River and in the Ohio River watershed (see Tiemann, Lawlis & Douglass, 2018).

Previous molecular characterizations of Forms A, B, and C yielded limited phylogenetic support for monophyly of each of the forms (Lee et al., 2005; Hedtke et al., 2008; Pigneur et al., 2014; Gomes et al., 2016). Based on our results for 1,699–30,027 nuclear genomic loci, the four invasive New World *Corbicula* forms represent highly distinct monophyletic nuclear lineages, although they have apparently engaged in multiple mt genome capture events through egg parasitism (Lee et al., 2005; Hedtke et al., 2008; Tiemann et al., 2017). Across our phylogenomic dataset, analyses showed limited evidence of nuclear genomic admixture among the invasive clones including locations where clones co-occur, A, B, and D in the Illinois River, A and B in the Rio Grande River, and A and C in La Plata Argentina (see Table S1; Fig. 2A). The only exception was Iguazu Falls Form C individual COR3–6 (Table S4) that had 2% assignment to Form B (Fig. 4E). This is consistent with previous 28S results for this same individual showing a heterozygous Form C/Form B 28S genotype (see Lee et al., 2005), but note that this specimen yielded a low number of homologous loci (Table S3) and that the 2% Form B assignment may be an underestimate. Our results indicate that egg parasitism and mt genome capture may be more prevalent among North American invasive Forms than nuclear genome hybridization and, together with previous studies using 28S sequences (see Lee et al., 2005) and microsatellite loci (see Pigneur et al., 2014), they raise the possibility that there is population level variation in how co-occurring forms interact genetically across their invasive range.

The phylogenomic data reinforce the utility of shell phenotype variation in identifying the four invasive New World *Corbicula* lineages and in monitoring their spread into new watersheds (Fig. 1). Form D’s most distinctive characteristics are the fine rust-colored rays radiating out from the umbo on the exterior of the shell and the creamy white nacre and purple lateral teeth on the interior (Tiemann et al., 2017). To-date, this phenotype combination has not been recorded for any of the other known New World or European (L-M Pigneur, 2019, personal communication) invasive forms although Britton & Morton (1986) reported an ostensibly similar *Corbicula* Asian morph “radiating streaks of orange brown on various parts of the shell exterior” that has yet to be genotyped.

Our phylogenomic analyses provided new insights into the phylogenetic relationships of the New World invasive lineages and the Lake Biwa endemic sexual species *C. sandai*. The modal topology, recovered from seven of nine phylogenomic datasets, placed *C. sandai* sister to a clade containing invasive Forms A–D (Fig. 3; Fig. S1). Previous studies using mt phylogenies recovered *C. sandai* nested among the invasive lineages (Lee et al., 2005; Hedtke et al., 2008; Hedtke, Glaubrecht & Hillis, 2011; Pigneur et al., 2014; Gomes et al., 2016), a topology also obtained in two of our nine phylogenomic datasets (Figs. S1C and S1I). In these two datasets, Form A clams placed sister to a clade containing invasive Forms B, C, and D and *C. sandai*. This discrepancy may have resulted from our designated outgroup, the estuarine *C. japonica*, having far fewer inferred homologous loci (26–229) compared to our ingroup samples (372–24,443; Table S3) complicating the determination of ancestral and derived polymorphisms (reviewed by Lyons-Weiler, Hoelzer & Tausch, 1998). In addition, the Structure analysis showed the partial assignment of some *C. sandai* individuals to multiple invasive lineages, indicating genotypic similarity in these comparisons, a factor that could have influenced its placement in the phylogenomic trees. Moreover, a recent DAPC analysis found a close relationship between Form A individuals and *C. japonica* using 10 nuclear microsatellite loci (Pigneur et al., 2014). Nevertheless, the weight of overall evidence in our genomic dataset favors a sister lineage status for *C. sandai* to the invasive lineages: it is not only the predominant phylogenomic placement recovered, but the mPTP, Structure, and DAPC analyses (Figs. 3–5; Figs. S1 and S3) all supported the distinctiveness of the Lake Biwa sexual endemic relative to the four invasive New World lineages.

Invasive New World Forms A, B, and C clams are known to be androgenetic clones as evidenced by a lack of complete meiotic metaphases, having unreduced spermatozoa, biflagellate sperm (Burch, Park & Chung, 1998; Lee et al., 2005; Hedtke et al., 2008; Pigneur et al., 2014) and can vary in their number of complete chromosomes, with triploid arrangements documented for Forms A and B (Burch, Park & Chung, 1998). Although the specific reproductive mode of Form D clams remains to be determined, its genomic profile, along with those for Forms A and B, is consistent with the expectations of clonality (see Table S1; Figs. 4 and 6; Fig. S5) including exhibiting departures from HWE expectations (Douhovnikoff & Leventhal, 2016), little to no genotypic diversity (Balloux, Lehmann & De Meeûs, 2003; Roman & Darling, 2007; Dybdahl & Drown, 2011), and high levels of heterozygote excess (Balloux, Lehmann & De Meeûs, 2003; Stoeckel et al., 2006). Other studies have also shown very little genetic variation across the New World and European invasive ranges for *Corbicula* Forms A, B, and C using mt COI (Lee et al., 2005; Pigneur et al., 2014; Gomes et al., 2016) and nuclear 28S ribosomal sequences (Lee et al., 2005) and nuclear microsatellite markers (Pigneur et al., 2014). These characteristically low diversity population genetic profiles could be a result of their clonal reproduction, compounded by convergent egg parasitism in the case of mt genomes, and/or founder effects.

Interestingly, the *F*_IS_ values obtained for Form A individuals varied across its New World range with some loci showing values closer to those of the sexually reproducing *C. sandai* (see Fig. 6C). Studies have found that this variability in *F*_IS_ values potentially indicates genotypes that are the result of rare sexual reproduction events (Balloux, Lehmann & De Meeûs, 2003; Halkett, Simon & Balloux, 2005; Vandepitte et al., 2010; Stoeckel & Masson, 2014). If this is the case for Form A individuals, rare sexual reproduction may have circumvented the accumulation of deleterious mutations enhancing its fitness and enabled it to rapidly spread across North and South America. Further sampling is clearly needed.

Our genomic results have implications for the origin of androgenesis in the genus *Corbicula*. Forms A, B, C, and D formed a distinct clonal clade in the majority (7/9) of our phylogenomic trees and the mPTP analysis indicating an apparent single origin. If that is indeed the case, it gives us a potential maximum age of 1.49 my (0.401–2.955 my highest posterior density) for the origin of this clade and potentially also for the origin of androgenesis in this genus. This is older than estimates for most other asexual clades, e.g., the New Zealand Mud Snail *Potamopyrgus antipodarum* (<0.50 my; Neiman, Jokela & Lively, 2005) and *Timema* walkingsticks (<0.50 my; Law & Crespi, 2002). For each of the four New World invasive lineages, their estimated ages range from 0.27 to 0.44 my, however, these are likely underestimates because they do not include information from their Old World populations of origin (see Lee et al., 2005; Hedtke et al., 2008; Hedtke, Glaubrecht & Hillis, 2011; Pigneur et al., 2014; Gomes et al., 2016). More sampling and a phylogenomic tree more representative for the entire genus is clearly necessary.

Within the four invasive New World lineages, our population-level analyses using Structure and DAPC showed no additional genetic structuring. Notably, Form A clustered into a single population despite ranging from Michigan to Patagonia. In contrast, our samples of the sexually reproducing Lake Biwa endemic *C. sandai* clustered into two distinct population groups with 5/6 individuals having ~100% assignment. Additional population variation within *C. sandai* is likely as many of the species groups endemic to Lake Biwa exhibit high degrees of genetic structuring (see Tabata et al., 2016; Miura et al., 2018). Further sampling is necessary to determine the population genetic structuring of *C. sandai*.

One surprising feature of our Structure results was that two of the six *C. sandai* individuals partially assigned to some or all of the New World invasive clonal Forms. One individual (CSA2–5) had 11% assignment to Form D, 5% to Form A, and less than 1% to Forms B and C whereas the other individual (CSA2–1) had 9% assignment to Form B, 3% to Form A, 2% to Form D, and less than 1% to Form C (see Fig. 4; Table S4). It is difficult with our dataset to distinguish whether this is a result of ancestral polymorphisms or partially successful androgenetic takeover events. This could be done for instance with ABBA–BABA tests (see Eaton & Ree, 2013), but this would require a more extensive sampling of *C. sandai* and most importantly an outgroup with a much higher share of homologous loci than we have available to us for *C. japonica*.

Our phylogenomic trees indicate that *C. sandai* and the invasive clonal lineages share a close common ancestor and thus likely share many plesiomorphic molecular characters. Likewise, other studies have postulated that the invasive clonal lineages originated from a shared ancestor with *C. sandai* (see Hedtke et al., 2008; Hedtke, Glaubrecht & Hillis, 2011) thereby potentially explaining these partial Structure assignments and their placement in the phylogenomic trees. Alternatively, if the partial assignment is a result of more recent genetic admixture, it has important consequences for the persistence of *C. sandai* in sympatry with invasive clones. Currently, with the exception of the estuarine *C. japonica*, known sexually reproducing lineages occur only in ancient freshwater lakes (e.g., *C. sandai*; Lee et al., 2005; Hedtke et al., 2008; Hedtke, Glaubrecht & Hillis, 2011; Pigneur et al., 2014) and are apparently missing from other lentic and lotic freshwater habitats where clonal lineages exclusively predominate (see Pigneur et al., 2014; Gomes et al., 2016). This raises the possibility that the androgenetic lineages have driven sexual congeners to extinction, possibly through egg parasitism (Hedtke et al., 2008), outside of ancient lakes. Invasive *Corbicula* forms do occur in Lake Biwa (Ishibashi & Komaru, 2003) and our Structure results may represent partial genomic incursions by the clonal lineages into the *C. sandai* genome. Simulation studies predict that in cases where androgenetic and sexual populations co-occur, androgenesis should quickly become fixed (see McKone & Halpern, 2003; summarized by Hedtke, Glaubrecht & Hillis, 2011; Pigneur et al., 2012). However, if these partial genomic assignments do represent genomic incursions of clonal lineages into *C. sandai* individuals, they were not completely successful indicating that *C. sandai* and other surviving sexual species may have some capacity to resist full androgenetic takeover. Why such a capacity would be restricted to species inhabiting ancient lake environments remains to be determined.

## Conclusions

Our phylogenomic results of 1,699–30,027 nuclear genomic loci showed the putative Form D reported by Tiemann et al. (2017) is a novel invasive *Corbicula* lineage, with a population genomic profile consistent with clonality, increasing the number of New World invasive lineages to four; Forms A, B, C, and D. These four lineages are readily identifiable based on their distinctive shell phenotypes, reinforcing the utility of their phenotypes in monitoring their spread into new watersheds. Our analyses recovered evidence that Forms A, B, C, and D comprised a distinct clonal clade, sister to *C. sandai*, with an estimated age of 1.49 my (± 0.401–2.955 my). We recovered limited evidence of nuclear genome interaction among the four invasive lineages but some *C. sandai* individuals displayed partial nuclear genomic Structure assignments with multiple invasive lineages.

## Supplemental Information

10.7717/peerj.7484/supp-1 Supplemental Information 1 Phylogenomic analyses confirm a novel invasive North American *Corbicula* (Bivalvia: Cyrenidae) lineage.

## Additional Information and Declarations

### DNA Deposition

The following information was supplied regarding the deposition of DNA sequences: Data is available at SRA: PRJNA526860.

### Data Availability

The following information was supplied regarding data availability: Specimens from this study are archived at the University of Michigan’s Museum of Zoology; UMMZ#s 266689, 300014, 300016–17, 300023, 300027–29, 305020–22, 305052, 305054. Detailed sampling information can be found in Table S1. Dryad: DOI 10.5061/dryad.4395r55.
