# Supplementary material for: Phylogenomic analyses confirm a novel invasive North American Corbicula (Bivalvia: Cyrenidae) lineage
Source: PeerJ. 2019 Aug 22;7:e7484. doi: 10.7717/peerj.7484 (PMC6708575; doi:10.7717/peerj.7484)
Supplement: Supplemental Information 1 [file peerj-07-7484-s001.docx]

**Supplementary information for:**

**Phylogenomic analyses confirm a novel invasive North American *Corbicula* (Bivalvia: Cyrenidae) lineage**

Amanda E. Haponski, Diarmaid Ó Foighil

University of Michigan, Department of Ecology and Evolutionary Biology and Museum of Zoology Division of Mollusks, Ann Arbor, MI 48109

**Appendix 1:**

Illumina sequencing returned raw read numbers ranging from 125,580 – 5,883,062 across the 36 corbiculid samples, with 12 individuals having fewer than 1,000,000 reads (Table S2). Clustering at 85, 90, and 95% similarity thresholds resulted in congruent numbers of loci across the 36 individuals that passed quality filtering. In general, the overall number of loci increased across the three similarity thresholds presumably due to homologous loci being classified as multiple loci at higher stringency thresholds (90 and 95%). The mean coverage depth of loci ranged from 13.2-104.0 for the 85% threshold, 13.2–101.7 for 90%, and 13.0–97.0 for 95%, with a Form C (COR3-6) and a *C. sandai* (CSA2-5) individual having the lowest coverage and a Form B (COR-6) and a Form D (COR-14) clam having the highest (Table S2).

We identified 1,699-30,027 nuclear genomic loci across the nine ddRADseq datasets for *Corbicula* clams. The number of homologous loci in the final datasets increased as the minimum number of individuals (75%, 50%, 25%) required for retaining a locus decreased. For the 85% threshold across 75% of the 36 samples (*N*=27 individuals), 2,181 loci were recovered in the final ddRADseq dataset whereas the number of loci for the 90 and 95% levels were 2,245 and 1,699, respectively. Decreasing the minimum taxon coverage from 75% to 50% (*N*=18 individuals) resulted in a five-fold increase in the number of loci: 9,166 for 85%, 9,901 for 90%, and 9,673 for 95% threshold datasets. The number of loci also increased when only 25% (*N*=9 individuals) of individuals was required to retain a locus (85% - 24,010, 90% - 26,768, 95% - 30,027). Higher numbers of loci were recovered in the invasive *Corbicula* morphotypes compared to the sexually reproducing outgroup samples. *Corbicula japonica* had the lowest numbers of homologous loci included in the alignments compared to the four invasive Forms (Table S3). *Corbicula sandai* had more homologous loci than *C. japonica,* but the number of loci was lower than those included for the clonal forms. Within the four clonal *Corbicula* forms, individuals had similar numbers of loci across all samples and across the different similarity thresholds and taxon coverages (Table S3).

**Table S1 Sampling description of *Corbicula* individuals used in this study.** Values include sampling locations with latitude and longitude, University of Michigan’s Museum of Zoology (UMMZ) Mollusk division catalog number, number of individuals, observed (*H*_O_) and expected (*H*_E_) heterozygosities, and the inbreeding coefficient (*F*_IS_). See Figs. 1 and 6 for map and boxplot of values.

| Taxon | Location | Latitude | Longitude | UMMZ | *N* |  | *H*_O_ | *H*_E_ | *F*_IS_ |
| --- | --- | --- | --- | --- | --- | --- | --- | --- | --- |
| Form A | -- | -- | -- |  | 13 |  | 0.113 ± 0.029 | 0.063 ± 0.015 | -0.798 ± 0.172 |
|  | Davis Ck., MI | 42.462°N | 83.708°W | 300014 | 2 |  |  |  |  |
|  | Illinois R., IL | 41.316°N | 88.692°W | 305020 | 5 |  |  |  |  |
|  | Rio Grande R., NM | 32.291°N | 106.826°W | 300016 | 2 |  |  |  |  |
|  | Panama Canal, Panama | 9.116°N | 79.697°W | 300023 | 2 |  |  |  |  |
|  | La Plata, Argentina | 34.960°S | 57.777°W | 300028 | 2 |  |  |  |  |
|  |  |  |  |  |  |  |  |  |  |
| Form B | -- | -- | -- |  | 7 |  | 0.124 ± 0.031 | 0.070 ± 0.016 | -0.771 ± 0.165 |
|  | Illinois R., IL | 41.316°N | 88.692°W | 305021 | 5 |  |  |  |  |
|  | Rio Grande R., NM | 32.291°N | 106.826°W | 300017 | 2 |  |  |  |  |
|  |  |  |  |  |  |  |  |  |  |
| Form C | -- | -- | -- |  | 3 |  | -- | -- | -- |
|  | Iguazu Falls, Argentina | 25.694°S | 54.427°W | 300027 | 1 |  |  |  |  |
|  | La Plata Argentina | 34.960°S | 57.777°W | 300029 | 2 |  |  |  |  |
|  |  |  |  |  |  |  |  |  |  |
| Form D | Illinois R., IL | 41.316°N | 88.692°W | 305022 | 5 |  | 0.147 ± 0.033 | 0.084 ± 0.017 | -0.757 ± 0.143 |
|  |  |  |  |  |  |  |  |  |  |
| *C. sandai* | -- | -- | -- |  | 6 |  | 0.117 ± 0.019 | 0.144 ± 0.019 | 0.185 ± 0.077 |
|  | Seta R., Lake Biwa, Japan | 34.975°N | 135.907°E | 305054 | 5 |  |  |  |  |
|  | Lake Biwa | -- | -- | 266689 | 1 |  |  |  |  |
|  |  |  |  |  |  |  |  |  |  |
| *C. japonica* | Natori R., Sendai Japan | 38.189°N | 140.939°E | 305052 | 2 |  | -- | -- | -- |

**Table S2** **Summary of ddRADseq data for each *Corbicula* individual sequenced including the number of raw reads, number of loci passing the paralog filter with a sequencing depth >6, and mean depth of loci with sequencing depth >6 for 85, 90, and 95% similarity thresholds.**

|  |  |  | 85% similarity | |  | 90% similarity | |  | 95% similarity | |
| --- | --- | --- | --- | --- | --- | --- | --- | --- | --- | --- |
| Taxon | Sample ID | Raw reads | loci | depth |  | loci | depth |  | loci | depth |
| Form A | COR1 | 1,505,604 | 30,191 | 34.1 |  | 32,719 | 33.3 |  | 36,623 | 31.4 |
|  | COR2 | 1,691,645 | 30,672 | 37.8 |  | 33,281 | 36.9 |  | 37,295 | 34.9 |
|  | COR3 | 4,489,392 | 44,849 | 74.8 |  | 48,632 | 71.5 |  | 54,526 | 66.4 |
|  | COR4 | 1,862,856 | 31,110 | 38.4 |  | 33,839 | 37.4 |  | 37,900 | 35.3 |
|  | COR5 | 3,838,302 | 41,034 | 68.9 |  | 44,648 | 67.5 |  | 51,283 | 63.0 |
|  | COR2-2 | 2,769,736 | 37,586 | 51.8 |  | 40,555 | 51.1 |  | 46,135 | 48.3 |
|  | COR2-3 | 1,716,000 | 33,657 | 35.2 |  | 35,986 | 34.4 |  | 39,965 | 32.9 |
|  | COR2-4 | 1,376,335 | 31,829 | 29.3 |  | 34,146 | 29.0 |  | 38,187 | 27.8 |
|  | COR2-5 | 1,524,015 | 35,060 | 27.8 |  | 37,600 | 26.8 |  | 41,524 | 26.4 |
|  | COR2-7 | 1,365,669 | 32,175 | 28.9 |  | 34,473 | 28.5 |  | 38,412 | 27.5 |
|  | COR2-9 | 749,464 | 19,360 | 19.5 |  | 20,642 | 18.6 |  | 21,982 | 19.0 |
|  | COR2-10 | 1,087,083 | 28,662 | 25.4 |  | 30,633 | 25.1 |  | 34,106 | 24.2 |
|  | COR2-11 | 572,266 | 12,604 | 24.6 |  | 13,248 | 24.4 |  | 13,974 | 23.9 |
|  |  |  |  |  |  |  |  |  |  |  |
| Form B | COR6 | 5,883,062 | 42,069 | 104.0 |  | 45,814 | 101.7 |  | 52,533 | 94.5 |
|  | COR7 | 3,463,831 | 44,771 | 56.3 |  | 48,457 | 54.8 |  | 54,266 | 51.9 |
|  | COR8 | 2,898,114 | 33,678 | 64.2 |  | 36,760 | 62.5 |  | 41,345 | 57.9 |
|  | COR9 | 2,585,613 | 33,123 | 57.0 |  | 36,088 | 55.5 |  | 40,404 | 51.5 |
|  | COR10 | 2,644,859 | 33,618 | 57.7 |  | 36,778 | 56.1 |  | 41,480 | 51.9 |
|  | COR3-1 | 1,922,510 | 33,316 | 42.4 |  | 35,851 | 41.8 |  | 40,349 | 39.4 |
|  | COR3-3 | 1,663,612 | 31,340 | 36.2 |  | 33,640 | 35.1 |  | 37,117 | 33.5 |
|  |  |  |  |  |  |  |  |  |  |  |
| Form C | COR3-6 | 331,884 | 6,784 | 13.2 |  | 7,014 | 13.4 |  | 7,101 | 13.7 |
|  | COR4-2 | 480,327 | 18,147 | 15.9 |  | 19,130 | 15.8 |  | 20,578 | 15.3 |
|  | COR4-3 | 1,822,633 | 27,256 | 42.9 |  | 29,435 | 40.4 |  | 32,858 | 39.5 |
|  |  |  |  |  |  |  |  |  |  |  |
| Form D | COR11 | 2,631,118 | 29,903 | 65.1 |  | 32,279 | 63.7 |  | 35,846 | 59.8 |
|  | COR12 | 2,415,662 | 29,399 | 61.3 |  | 31,803 | 59.9 |  | 35,277 | 56.1 |
|  | COR13 | 2,860,612 | 29,715 | 74.1 |  | 32,205 | 71.7 |  | 35,871 | 67.5 |
|  | COR14 | 5,004,516 | 36,163 | 102.7 |  | 39,049 | 101.7 |  | 44,174 | 97.0 |
|  | COR15 | 4,249,969 | 51,015 | 57.6 |  | 55,441 | 56.1 |  | 62,160 | 54.2 |
|  |  |  |  |  |  |  |  |  |  |  |
| *C. sandai* | CSA1 | 864,701 | 21,509 | 24.7 |  | 22,705 | 24.9 |  | 24,478 | 24.6 |
|  | CSA2-1 | 903,797 | 21,998 | 28.5 |  | 23,498 | 28.3 |  | 25,774 | 27.3 |
|  | CSA2-2 | 125,580 | 3,881 | 14.3 |  | 4,012 | 14.7 |  | 4,057 | 14.5 |
|  | CSA2-3 | 135,134 | 3,144 | 15.2 |  | 3,273 | 15.5 |  | 3,378 | 16.4 |
|  | CSA2-4 | 162,484 | 5,382 | 14.4 |  | 5,568 | 14.4 |  | 5,647 | 14.2 |
|  | CSA2-5 | 184,407 | 6,648 | 13.2 |  | 6,913 | 13.2 |  | 7,027 | 13.0 |
|  |  |  |  |  |  |  |  |  |  |  |
| *C. japonica* | CJA2-3 | 625,320 | 18,083 | 22.0 |  | 19,529 | 22.4 |  | 21,688 | 21.7 |
|  | CJA2-4 | 601,456 | 15,058 | 22.9 |  | 16,341 | 23.5 |  | 18,050 | 24.0 |

**Table S3 Summary of the final number of ddRADseq loci for each *Corbicula* individual for the 85, 90, and 95% similarity thresholds and 75, 50, and 25% taxon coverage datasets.**

|  |  | 85% similarity | | |  | 90% similarity | | |  | 95% similarity | | |
| --- | --- | --- | --- | --- | --- | --- | --- | --- | --- | --- | --- | --- |
| Taxon | Sample ID | 75% | 50% | 25% |  | 75% | 50% | 25% |  | 75% | 50% | 25% |
| Form A | COR1 | 2,160 | 8,432 | 16,808 |  | 2,223 | 9,164 | 18,876 |  | 1,689 | 9,026 | 21,230 |
|  | COR2 | 2,168 | 8,526 | 17,027 |  | 2,232 | 9,227 | 19,079 |  | 1,693 | 9,096 | 21,499 |
|  | COR3 | 2,139 | 8,570 | 18,130 |  | 2,217 | 9,339 | 20,397 |  | 1,685 | 9,232 | 23,018 |
|  | COR4 | 2,155 | 8,456 | 16,945 |  | 2,224 | 9,209 | 19,052 |  | 1,691 | 9,052 | 21,431 |
|  | COR5 | 2,141 | 8,839 | 19,364 |  | 2,213 | 9,645 | 21,705 |  | 1,690 | 9,523 | 24,443 |
|  | COR2-2 | 2,168 | 8,966 | 19,480 |  | 2,235 | 9,705 | 21,732 |  | 1,697 | 9,539 | 24,396 |
|  | COR2-3 | 2,156 | 8,374 | 16,892 |  | 2,221 | 9,080 | 18,854 |  | 1,684 | 8,935 | 21,246 |
|  | COR2-4 | 2,173 | 8,628 | 17,903 |  | 2,235 | 9,341 | 19,945 |  | 1,696 | 9,176 | 22,430 |
|  | COR2-5 | 2,134 | 8,013 | 15,609 |  | 2,195 | 8,715 | 17,399 |  | 1,673 | 8,574 | 19,642 |
|  | COR2-7 | 2,171 | 8,767 | 18,324 |  | 2,235 | 9,486 | 20,433 |  | 1,695 | 9,324 | 22,937 |
|  | COR2-9 | 1,635 | 4,088 | 6,511 |  | 1,712 | 4,455 | 7,239 |  | 1,329 | 4,296 | 7,888 |
|  | COR2-10 | 2,160 | 8,340 | 16,650 |  | 2,223 | 9,026 | 18,549 |  | 1,689 | 8,863 | 20,877 |
|  | COR2-11 | 1,343 | 2,967 | 4,372 |  | 1,395 | 3,193 | 4,803 |  | 1,116 | 3,065 | 5,102 |
|  |  |  |  |  |  |  |  |  |  |  |  |  |
| Form B | COR6 | 2,169 | 8,276 | 14,914 |  | 2,237 | 8,910 | 16,329 |  | 1,696 | 8,614 | 16,856 |
|  | COR7 | 2,171 | 8,128 | 14,225 |  | 2,234 | 8,767 | 15,567 |  | 1,689 | 8,483 | 16,103 |
|  | COR8 | 2,174 | 8,200 | 13,698 |  | 2,243 | 8,831 | 15,035 |  | 1,697 | 8,541 | 15,534 |
|  | COR9 | 2,169 | 8,157 | 13,502 |  | 2,235 | 8,760 | 14,801 |  | 1,697 | 8,475 | 15,301 |
|  | COR10 | 2,174 | 8,157 | 13,520 |  | 2,242 | 8,764 | 14,807 |  | 1,693 | 8,465 | 15,328 |
|  | COR3-1 | 2,174 | 8,164 | 14,079 |  | 2,240 | 8,773 | 15,370 |  | 1,689 | 8,489 | 15,899 |
|  | COR3-3 | 2,130 | 7,397 | 11,773 |  | 2,200 | 7,976 | 12,859 |  | 1,667 | 7,702 | 13,241 |
|  |  |  |  |  |  |  |  |  |  |  |  |  |
| Form C | COR3-6 | 533 | 835 | 1,187 |  | 547 | 890 | 1,297 |  | 372 | 684 | 1,128 |
|  | COR4-2 | 1,776 | 4,207 | 6,646 |  | 1,818 | 4,441 | 7,122 |  | 1,345 | 3,867 | 6,849 |
|  | COR4-3 | 1,772 | 4,400 | 7,179 |  | 1,832 | 4,649 | 7,749 |  | 1,368 | 4,104 | 7,594 |
|  |  |  |  |  |  |  |  |  |  |  |  |  |
| Form D | COR11 | 2,155 | 6,710 | 11,610 |  | 2,225 | 7,158 | 12,617 |  | 1,684 | 6,563 | 12,849 |
|  | COR12 | 2,153 | 6,668 | 11,531 |  | 2,222 | 7,129 | 12,563 |  | 1,683 | 6,529 | 12,764 |
|  | COR13 | 2,154 | 6,727 | 11,659 |  | 2,221 | 7,175 | 12,693 |  | 1,681 | 6,576 | 12,889 |
|  | COR14 | 2,149 | 6,782 | 12,585 |  | 2,224 | 7,243 | 13,647 |  | 1,683 | 6,665 | 13,859 |
|  | COR15 | 2,124 | 6,608 | 12,113 |  | 2,208 | 7,100 | 13,264 |  | 1,677 | 6,597 | 13,640 |
|  |  |  |  |  |  |  |  |  |  |  |  |  |
| *C. sandai* | CSA1 | 1,608 | 3,944 | 6,762 |  | 1,583 | 4,008 | 7,151 |  | 1,096 | 3,245 | 6,529 |
|  | CSA2-1 | 1,910 | 4,822 | 7,749 |  | 1,943 | 5,012 | 8,295 |  | 1,410 | 4,359 | 8,052 |
|  | CSA2-2 | 497 | 869 | 1,246 |  | 486 | 879 | 1,286 |  | 323 | 678 | 1,136 |
|  | CSA2-3 | 419 | 732 | 1,028 |  | 420 | 751 | 1,061 |  | 289 | 573 | 933 |
|  | CSA2-4 | 688 | 1,190 | 1,800 |  | 680 | 1,218 | 1,867 |  | 451 | 939 | 1,655 |
|  | CSA2-5 | 894 | 1,636 | 2,376 |  | 920 | 1,719 | 2,496 |  | 644 | 1,400 | 2,280 |
|  |  |  |  |  |  |  |  |  |  |  |  |  |
| *C. japonica* | CJA2-3 | 102 | 171 | 229 |  | 84 | 138 | 178 |  | 28 | 55 | 74 |
|  | CJA2-4 | 98 | 152 | 194 |  | 77 | 117 | 149 |  | 26 | 47 | 66 |

**Table S4 Population assignment values for the four invasive *Corbicula* lineages and *C. sandai* individuals from the Structure analyses.** Information for each individual includes taxon designation, individual id code, and proportion of assignment. See Fig. 4 for Structure bar graphs.

| Taxon | Individual | K1 (orange) | K2 (purple) | K3 (pink) | K4 (green) | K5 (blue) |
| --- | --- | --- | --- | --- | --- | --- |
| Form A | COR2-9 | 1.000 | 0.000 | 0.000 | 0.000 | 0.000 |
|  | COR3 | 1.000 | 0.000 | 0.000 | 0.000 | 0.000 |
|  | COR2-4 | 1.000 | 0.000 | 0.000 | 0.000 | 0.000 |
|  | COR5 | 1.000 | 0.000 | 0.000 | 0.000 | 0.000 |
|  | COR2-7 | 1.000 | 0.000 | 0.000 | 0.000 | 0.000 |
|  | COR2-3 | 1.000 | 0.000 | 0.000 | 0.000 | 0.000 |
|  | COR2-2 | 1.000 | 0.000 | 0.000 | 0.000 | 0.000 |
|  | COR2-10 | 1.000 | 0.000 | 0.000 | 0.000 | 0.000 |
|  | COR1b | 1.000 | 0.000 | 0.000 | 0.000 | 0.000 |
|  | COR2b | 1.000 | 0.000 | 0.000 | 0.000 | 0.000 |
|  | COR4b | 1.000 | 0.000 | 0.000 | 0.000 | 0.000 |
|  | COR2-5 | 1.000 | 0.000 | 0.000 | 0.000 | 0.000 |
|  | COR2-11 | 1.000 | 0.000 | 0.000 | 0.000 | 0.000 |
|  |  |  |  |  |  |  |
| Form B | COR3-3 | 0.000 | 1.000 | 0.000 | 0.000 | 0.000 |
|  | COR3-1 | 0.000 | 1.000 | 0.000 | 0.000 | 0.000 |
|  | COR7 | 0.000 | 1.000 | 0.000 | 0.000 | 0.000 |
|  | COR6 | 0.000 | 1.000 | 0.000 | 0.000 | 0.000 |
|  | COR8 | 0.000 | 1.000 | 0.000 | 0.000 | 0.000 |
|  | COR9 | 0.000 | 1.000 | 0.000 | 0.000 | 0.000 |
|  | COR10 | 0.000 | 1.000 | 0.000 | 0.000 | 0.000 |
|  |  |  |  |  |  |  |
| Form C | COR4-3 | 0.000 | 0.000 | 1.000 | 0.000 | 0.000 |
|  | COR4-2 | 0.000 | 0.000 | 1.000 | 0.000 | 0.000 |
|  | COR3-6 | 0.003 | 0.019 | 0.978 | 0.001 | 0.000 |
|  |  |  |  |  |  |  |
| Form D | COR13 | 0.000 | 0.000 | 0.000 | 1.000 | 0.000 |
|  | COR12 | 0.000 | 0.000 | 0.000 | 1.000 | 0.000 |
|  | COR11 | 0.000 | 0.000 | 0.000 | 1.000 | 0.000 |
|  | COR14 | 0.000 | 0.000 | 0.000 | 1.000 | 0.000 |
|  | COR15 | 0.000 | 0.000 | 0.000 | 1.000 | 0.000 |
|  |  |  |  |  |  |  |
| *C. sandai* | CSA2-4 | 0.000 | 0.000 | 0.000 | 0.000 | 1.000 |
|  | CSA2-2 | 0.000 | 0.000 | 0.000 | 0.000 | 1.000 |
|  | CSA1 | 0.000 | 0.000 | 0.000 | 0.000 | 1.000 |
|  | CSA2-3 | 0.000 | 0.000 | 0.001 | 0.001 | 0.998 |
|  | CSA2-5 | 0.048 | 0.002 | 0.009 | 0.113 | 0.828 |
|  | CSA2-1 | 0.029 | 0.086 | 0.001 | 0.023 | 0.861 |

**Figure S1**

1. 90-50


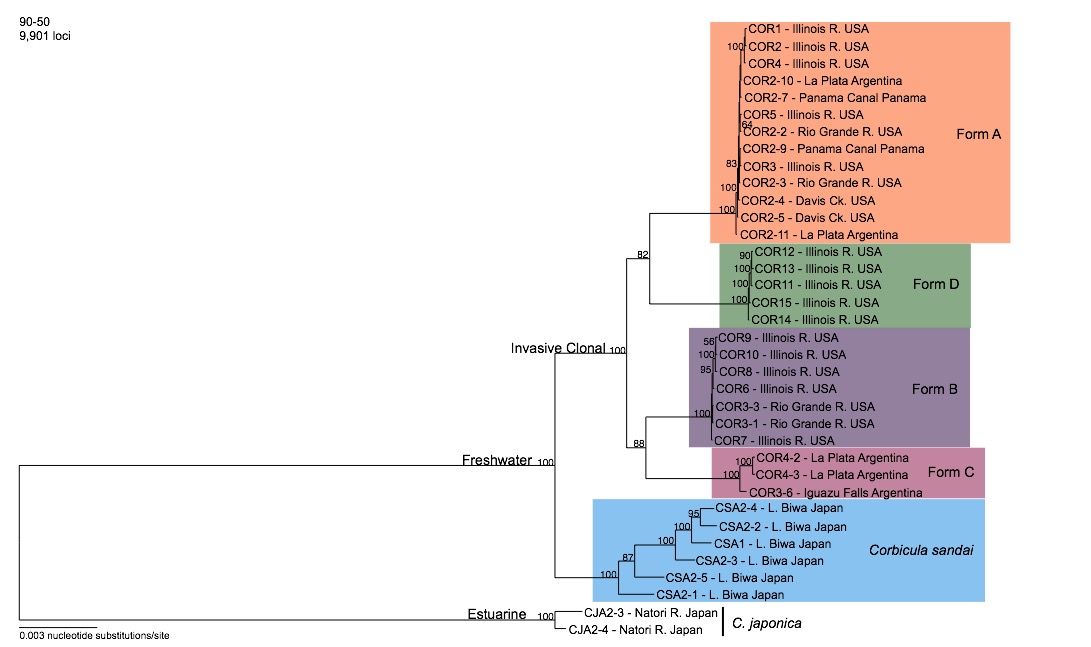


1. 90-25


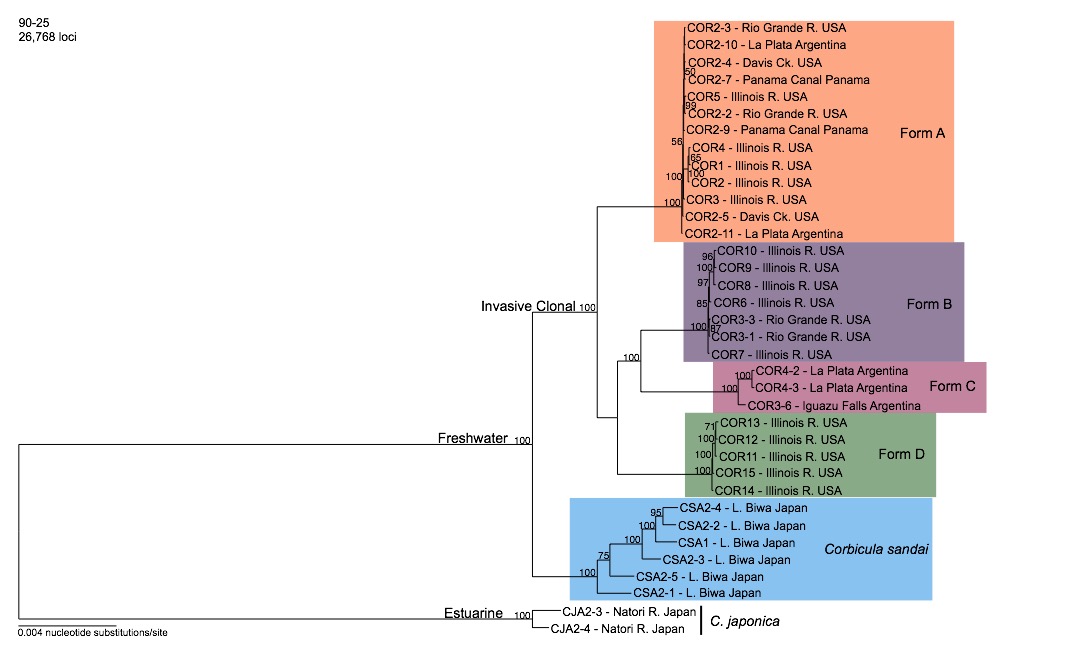


1. 85-75


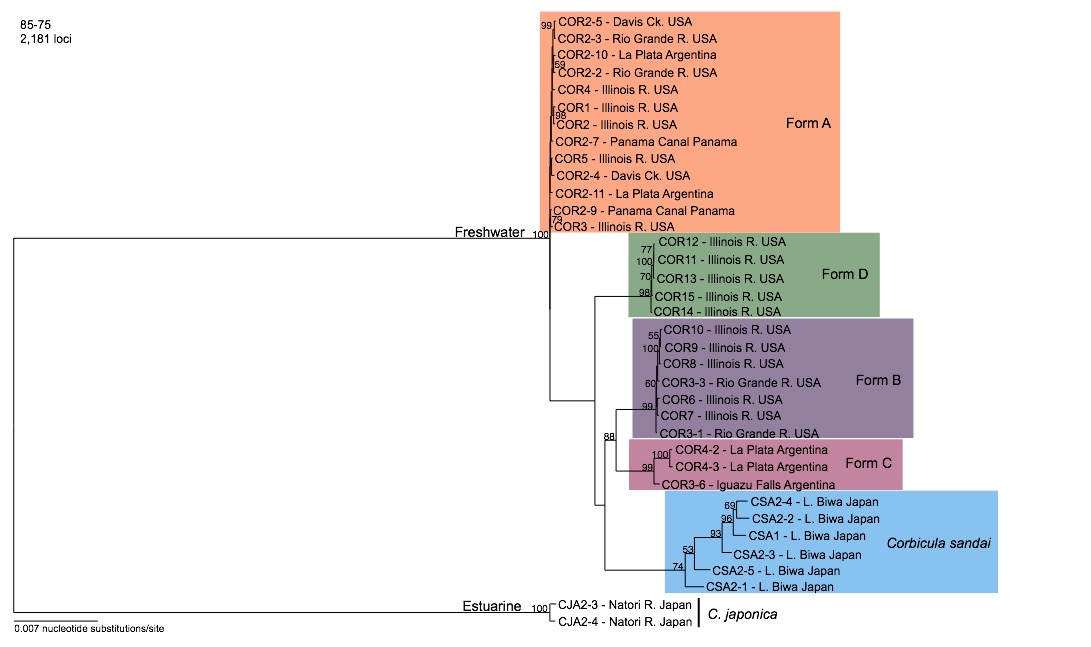


1. 85-50 with all individuals


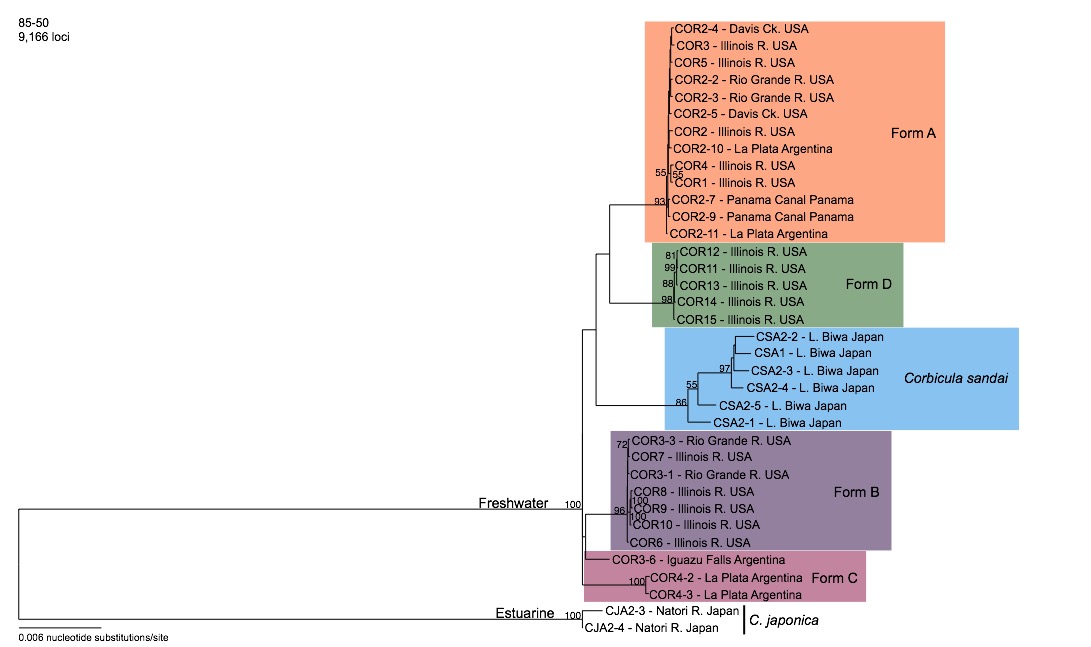


1. 85-50 with individuals dropped


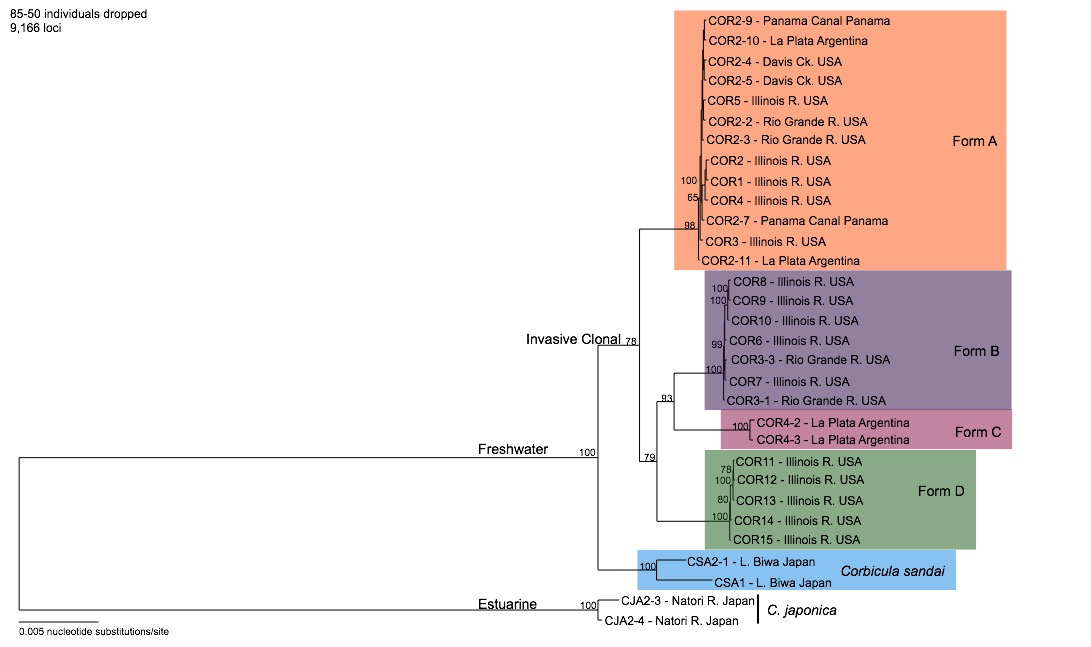


1. 85-25


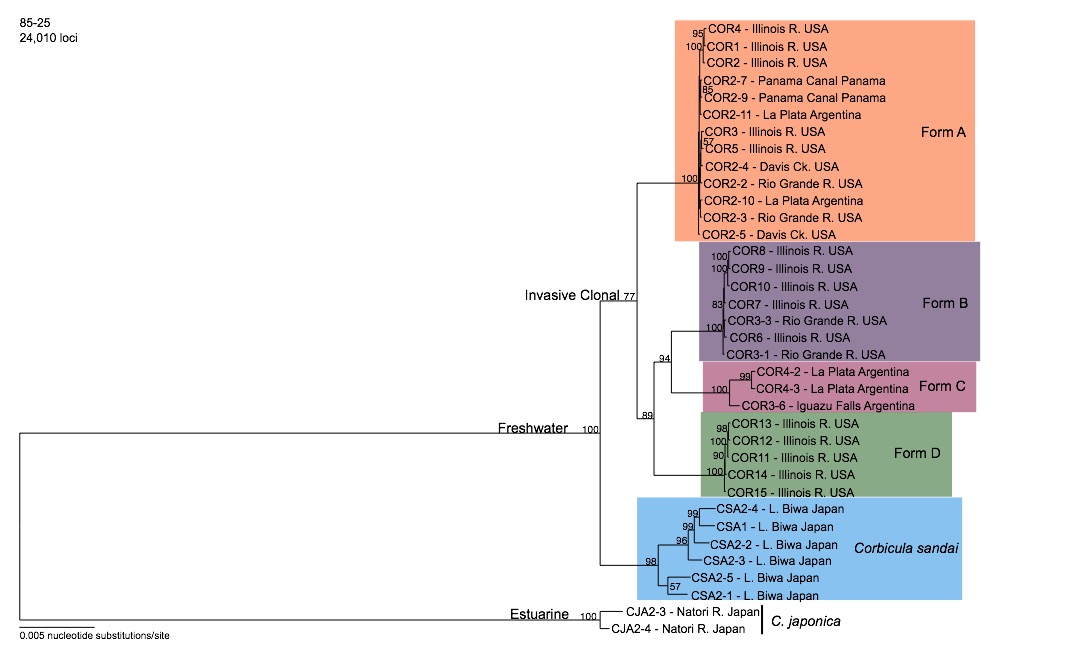


1. 95-75


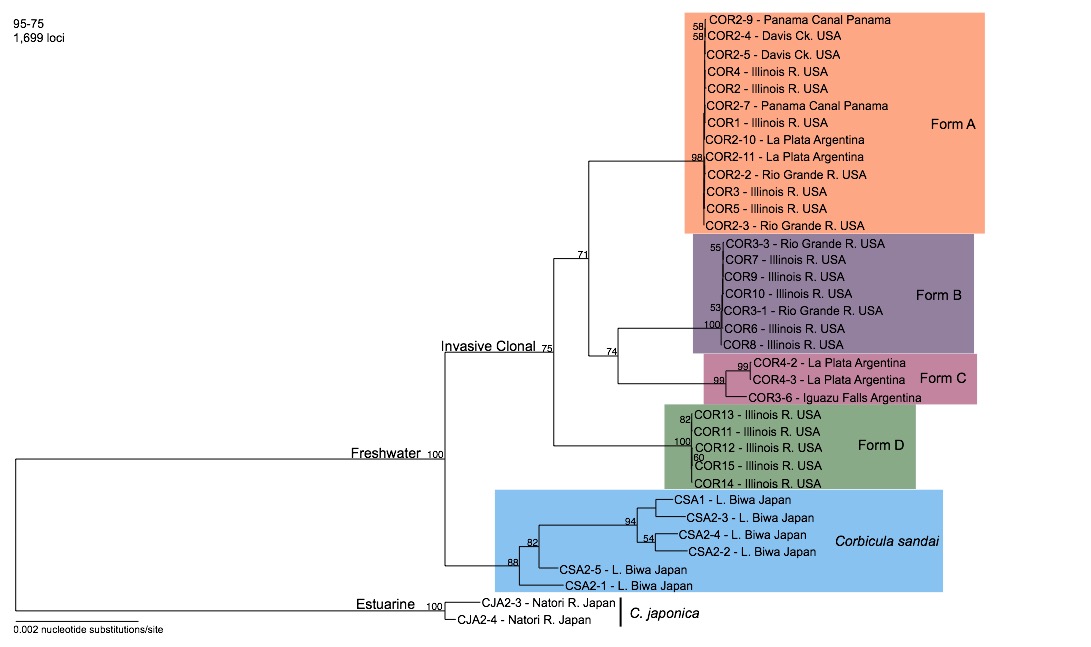


1. 95-50


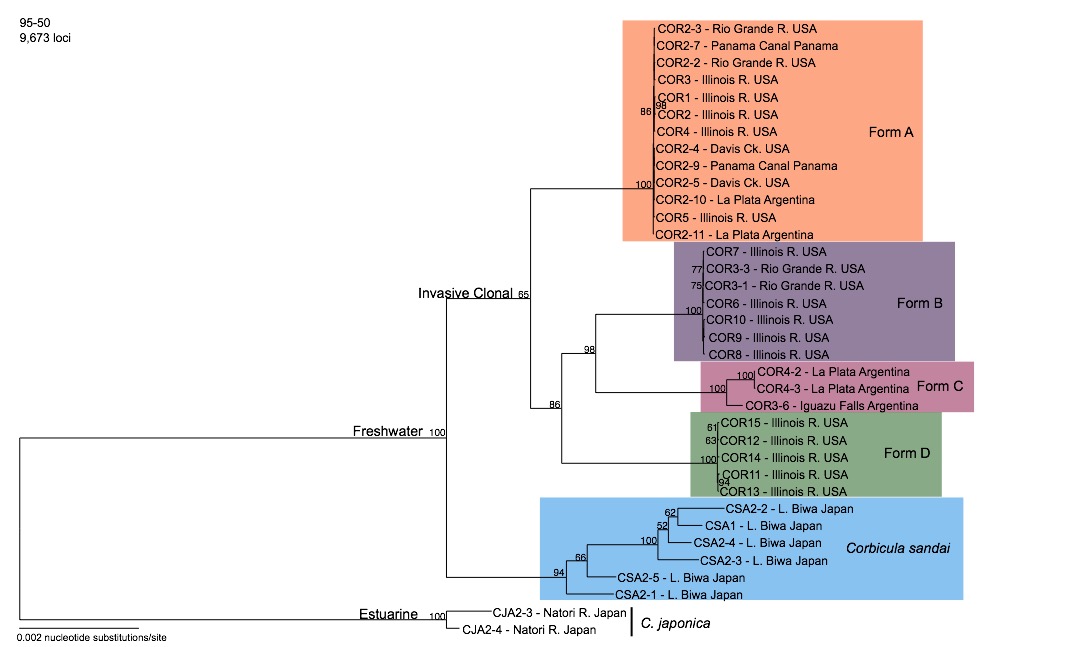


1. 95-25


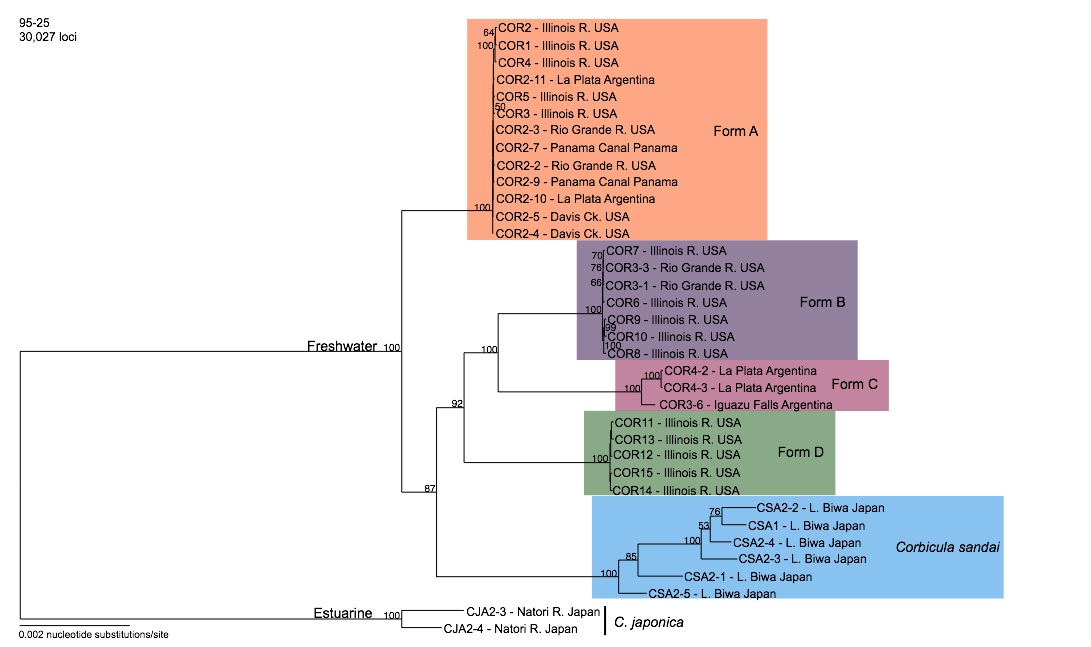


**Figure S1 Maximum likelihood phylogenomic trees depicting relationships among the sampled *Corbicula* individuals.** Individuals were clustered at three different similarity thresholds (85, 90, and 95%) and minimum taxon coverages (75, 50, and 25%). Trees depicted include (a) 90% similarity threshold, 50% of taxa, totaling 9,901 loci, (b) 90%, 25%, 26,768 loci, (c) 85%, 75%, 2,181 loci, (d) 85%, 50%, 9,166 loci, (e) 85%, 50% with individuals with low numbers of loci dropped, 9,166 loci, (f) 85%, 25%, 24,010 loci, (g) 95%, 75%, 1,699 loci, (h) 95%, 50%, 9,673 loci, and (i) 95%, 25%, 30,027 loci trees. Trees were rooted to the estuarine *C. japonica.* Values on tree nodes indicate bootstrap supports.

**Figure S2**

**Figure S2** **Scatter plots showing the (a) optimal number of principal components and (b) Bayesian Information Criterion (BIC) used to determine the most likely number of clusters in the dataset for the Discriminant Analysis of Principal Components (DAPC; *Jombart, Devillard, & Balloux, 2010*) of the four invasive *Corbicula* lineages and *C. sandai* clams.** See Fig. 5 for DAPC plot.

**Figure S3**

**Figure S3 Species limits for the invasive *Corbicula* lineages and *C. sandai* individuals based on MCMC mPTP analysis of the 90-75 RAxML gene tree constructed with 2,245 loci.** Branches are color‐coded to represent speciation (black) or coalescence (red) events. Values at nodes indicate probability of a speciation event based on mPTP MCMC analysis. Photos of Forms A, B, and D were taken by M. Jared Thomas and Danielle Ruffatto and Form C, C. sandai, and C. japonica by Taehwan Lee.

**Figure S4**

**Figure S4** **Scatter plots showing the most likely *K* for (a) the combined analysis including the four invasive *Corbicula* lineages and *C. sandai* individuals, (b) Form A, (c) Form B, (d) Form C, (e) Form D, and (f) *C. sandai* clams using the Δ*K* method of *Evanno et al. (2005)* and the mean natural log of the probability (+/- standard deviation (SD)) in Structure Harvester (*Earl & vonHoldt, 2012*).** See Fig. 4 for Structure bar graphs.

**Figure S5**

**Figure S5 Structure bar graphs showing the most likely assignment of Form A individuals for *K=*3, 4, and 5 population clusters (see Fig. 4 for *K*=2 and 6).** Structure analyses used a single SNP per locus (totaling 2,175 SNPs) for each individual and each vertical bar represents an individual clam. Labels on Structure graphs indicate the sampling locations for each individual (see Table S1). Each bar graph shows no additional population structuring for Form A despite samples spanning from North (Michigan) to South (Argentina) America.
